# Inoculation with cadmium/lead-tolerant bacteria enhances phytoremediation of *Amorpha fruticosa* L. by shifting key taxa and improving microbial stability

**DOI:** 10.1128/aem.00068-26

**Published:** 2026-05-15

**Authors:** Zexun Liu, Yugeng Guo, Yiming Fan, Changhui Geng, Abd Ur Rafy, Lele Zheng, Huihui Xu, Mingmao Sun, Xiangping Guo, Shaoge Jin

**Affiliations:** 1 Hohai University College of Agricultural Engineering and Science 626800, Nanjing, Jiangsu, China; 2 Zhenjiang Urban Water Conservancy Management Office, Zhenjiang, Jiangsu, China; 3 Shanghai Jiading District Riverway Sluice Management Office, Jiading, Shanghai, China; The University of Arizona, Tucson, Arizona, USA

**Keywords:** growth-promoting bacteria with cadmium and lead tolerance, *Amorpha fruticosa *L*.*, cadmium- and lead-contaminated soil, rhizosphere soil microbial community

## Abstract

The joint remediation of heavy metal (HM)-contaminated soil using beneficial microorganisms and plants has gained increasing attention as a sustainable approach. In this study, three growth-promoting bacterial strains (JG1, 2G5, and 2G6) with strong cadmium (Cd) and lead (Pb) tolerance were isolated through multistaged screening, and the effects and underlying mechanisms of soil HM remediation were investigated. Stress-resistant growth experiments confirmed the growth viability of these strains under Cd and Pb stress. The inoculation with the tolerant bacteria enhanced the biomass (by 13.71%–23.25%), plant tissue metal concentrations (by 16.07%–62.50%), and HM accumulation (by 41.04%–100.63%) in *Amorpha fruticosa* L. grown in contaminated soil, with 2G5 demonstrating the most pronounced effect. Principal coordinate analysis indicated that HM contamination exerted a greater impact on rhizosphere soil bacterial communities than did bacterial inoculation. Among the tested strains, 2G5 induced the most significant alterations in microbial composition. Furthermore, niche shifts in key taxa (e.g., p_*Patescibacteria* and g_*Flavisolibacter*) and enhanced microbial stability were identified as potential strategies for mitigating HM stress and promoting phytoextraction. Partial least squares path modeling revealed that the application of tolerance-promoting bacteria significantly reduced soil Cd risk through the regulation of soil nutrients, enzyme activity, and plant biomass, whereas Pb levels were primarily influenced by plant biomass and microbial diversity. Overall, this study provides an efficient strategy for remediating Cd- and Pb-contaminated soils through the synergistic application of specific microbial inoculants and *A. fruticosa* L.

**IMPORTANCE:**

HM contamination poses severe threats to ecosystem safety and human health. This study demonstrates that inoculating *Amorpha fruticosa* L. with cadmium/lead-tolerant plant growth-promoting bacteria (PGPB) (especially strain 2G5) significantly enhances phytoremediation efficiency by increasing plant biomass and metal accumulation. More importantly, we reveal that bacterial inoculation reshapes the rhizosphere microbial community, promotes niche shifts in key taxa (e.g., *Patescibacteria* and *Flavisolibacter*), and enhances microbial network stability, which collectively improve plant adaptability to metal stress. These findings provide a microbial-enhanced phytoremediation strategy that is sustainable and eco-friendly, offering practical insights for the remediation of HM-contaminated soils in real-world scenarios, especially in regions with leguminous vegetation.

## INTRODUCTION

Unregulated anthropogenic activities such as excessive mining, improper agricultural practices, and inadequate waste management have resulted in severe soil heavy metal (HM) contamination (1). As non-essential HMs and common soil pollutants, cadmium (Cd) and lead (Pb) are identified as priority contaminants by international regulatory agencies (2). Their high bioavailability and bio-accumulative characteristics facilitate their transfer and accumulation across the soil-microorganism-plant continuum, which poses significant risks to human health via the food chain, including renal dysfunction, cardiovascular disorders, and carcinogenesis (3, 4). Therefore, the development of effective management strategies and the selection of reliable remediation technologies for Cd- and Pb-contaminated soils are critical for environmental restoration.

Conventional remediation techniques for soil HMs, such as electrokinetic treatment, chemical stabilization, and thermal desorption, are frequently constrained by high operational costs and potential for secondary pollution (5, 6). As a promising alternative, phytoremediation utilizing plant-specific physiological traits offers a sustainable approach due to its environmental compatibility, economic feasibility, and ecological safety (7, 8). However, existing phytoremediation research predominantly focuses on metal hyperaccumulators (HMH), which often suffer from slow growth rates, limited biomass, and shallow rooting systems, thereby limiting their practical applications (9, 10). Recent studies have highlighted leguminous plants as emerging candidates for soil HM remediation, attracting great attention from researchers because of their robust biomass, effective net metal extraction capacity, and inherent tolerance to HMs (11). Consequently, the strategic (targeted) application of leguminous species may represent a viable phytoremediation pathway for remediating HM-contaminated soils.

*Amorpha fruticosa* L. is a deciduous shrub of the genus *Amorpha* within the Leguminosae family and exhibits strong environmental adaptability (e.g., drought resistance, nutrient-poor tolerance, salt tolerance, and HM tolerance) and contributes to soil fertility enhancement (12, 13). These attributes underscore its potential application in soil HM remediation. The introduction of beneficial microorganisms into plant rhizospheres is a widely adopted approach to enhance phytoextraction efficiency (14). However, the adaptability and ecological performance of microbial strains in contaminated environments remain uncertain and are largely influenced by the ecological conditions of their origin. Therefore, endophytes isolated from pioneer plants that have long been exposed to HM-contaminated environments may exhibit superior tolerance and adaptability to HMs, thereby contributing to the alleviation of environmental stress for host plants (15, 16). Our previous research has demonstrated that certain tolerant PGPB, such as *Paenibacillus polymyxa* WZ14 and *Trichoderma harzianum* J2, isolated from mining-affected soils, can significantly improve the biomass and Cd/Pb phytoextraction efficiency of leguminous species (*Sophora japonica* and *Leucaena leucocephala*) (17, 18). In view of theprevious research experience and the potential ability of *A. fruticosa* L., this study screened endophytic strains resistant to Cd and Pb from host plants growing in HM-contaminated areas for a long time and inoculated these strains into *A. fruticosa* L. to evaluate the remediation effect and HM extraction ability of *A. fruticosa* L. Therefore, further systematic investigation is imperative to evaluate the synergistic effects of *A. fruticosa* L. and tolerance-promoting bacteria, as well as to clarify the potential interactions between microorganisms and host plants.

In this study, three HM-tolerant PGPBs were selected based on their performance in HM stress-resistance assays and physiological and biochemical evaluations and subsequently inoculated into *A. fruticosa* L. for a duration of 5 months. Given the critical role of rhizosphere microflora in mediating plant-metal interactions, high-throughput 16S rRNA gene sequencing using the Illumina MiSeq platform was employed to investigate taxonomic shifts in soil bacterial communities under both initial and contaminated soil conditions. The objectives of this study were as follows: (i) to assess the effects of tolerance-promoting bacterial inoculation on plant biomass, soil properties (including nutrient availability and enzyme activities), and the accumulation of Cd and Pb in *A. fruticosa* L.; (ii) to elucidate the alterations in rhizosphere microbial community structure in response to bacterial inoculation and HM contamination; and (iii) to apply partial least squares path modeling (PLS-PM) to reveal the potential mechanisms by which tolerance-promoting bacteria influence Cd and Pb remediation in soil.

## MATERIALS AND METHODS

On 21 September 2021, a *Celosia argentea* L. plant growing near the tailings in the Jinding mining area (114°12′E, 39°21′N) in Duchang County, Jiujiang City, Jiangxi Province, was collected and transported to the Soil and Water Conservation Laboratory of Nanjing Forestry University for isolation of cadmium- and lead-tolerant PGPB.

Microbial isolation and purification procedures were conducted. After thorough cleaning and surface sterilization, the roots, stems, and leaves of the *C. argentea* L. were chopped into small sections and homogenized separately in a sterile mortar. A 1 mL aliquot of the homogenate was transferred into a 500 mL Erlenmeyer flask containing 400 mL of LB broth. After 2 days of incubation, the culture was serially diluted (10^−4^, 10^−5^, 10^−6^, and 10^−7^) to generate four bacterial suspensions. Subsequently, 1 mL of each dilution was spread on nutrient agar (NA, for bacterial culture) and potato dextrose agar (PDA, for fungal culture) plates, both free of HMs. Cultivation was conducted at 28°C in an incubator for 2 days (bacteria) and 5 days (fungi). Colonies that exhibited distinct morphologies were selected and subcultured. During subculturing, actidione and chloramphenicol were added to PDA and NA plates, respectively, to inhibit unwanted growth and purify the target strains. A total of 15 purified isolates (12 bacterial and 3 fungal strains) were obtained (Fig. S1). These strains were preserved in 50% sterile glycerol and stored at −80°C for further screening.

The 15 isolates were subsequently tested for HM tolerance by inoculation onto NA plates supplemented with 400 mg/L Pb^2+^ and 50 mg/L Cd^2+^ using the three-point method, followed by incubation at 28°C for 3 d. The colony diameters were recorded as indicators of growth performance under metal stress. Five Cd- and Pb-tolerant strains were identified based on this assay (Table S1. These strains were further subjected to physiological and biochemical characterization, including phosphate solubilization, nitrogen fixation, IAA production, siderophore production, and citric acid secretion, to evaluate their plant growth-promoting potential. Based on a comprehensive assessment (Table S2), three strains with strong growth-promoting traits and high HM tolerance were selected for subsequent experiments. The 16S rDNA sequences of these strains were determined (Fig. S2), and the strains were identified as *Serratia marcescens* JG1, *Serratia marcescens* 2G5, and *Achromobacter mucilens* 2G6.

### Stress-resistant growth experiment of HM-tolerant PGPB

The growth performance of tolerance-promoting bacterial strains under HM stress was quantitatively assessed using a resistance growth assay as described previously (18). Cadmium chloride (CdCl_2_·5H_2_O) and lead nitrate (Pb(NO_3_)_2_) were used to prepare LB media containing various concentrations of Cd²^+^ and Pb²^+^ , following the formulations listed in Table 1. Actively growing bacterial cultures (colony diameter ~1 mm) were inoculated into 500 mL Erlenmeyer flasks containing 400 mL of the prepared LB medium. LB medium without bacterial inoculum served as the control. Each treatment was performed in triplicate (18). The cultures were incubated in a shaking incubator (180 rpm, 25°C ± 0.3) for 72 h. The optical density at 600 nm (OD_600_) was measured at 6-h intervals (6, 12, 24, 36, 48, 60, and 72 h) using an ultraviolet spectrophotometer (UV-8000t, Shanghai Metash Instruments Co., Ltd.) to monitor the bacterial growth under different HM stress conditions.

**TABLE 1:** Concentration of HMs in culture solution

CK	Cd + Pb (mg/kg) 0 + 0		
HM treatment	Culture solution with a single HM		Culture solution with mixed HMs
1	50 ± 0	0 ± 400	50 ± 400
2	100 ± 0	0 ± 600	100 ± 600
3	150 ± 0	0 ± 800	150 ± 800
4	200 ± 0	0 ± 1000	200 ± 1000
5	250 ± 0	0 ± 1500	250 ± 1500
6	300 ± 0	0 ± 2000	300 ± 2000

### Experimental materials

The experimental plant species used in this study was *Amorpha fruticosa* L., obtained in March 2022 from Yifeng Seed Industry Co., Ltd., China. Prior to the pot experiment, the seeds were surface-sterilized. The test soil was collected from the 0 to 20 cm topsoil layer of farmland located at the Baguazhou experimental site (118°50′E, 32°11′N) in the Qixia District, Nanjing. The soil was sieved through a 5 mm × 5 mm mesh to remove stones and plant debris. To simulate contamination, 15 L of a mixed aqueous solution containing cadmium chloride (CdCl_2_·5H_2_O) and lead nitrate (Pb(NO_3_)_2_) at final concentrations of approximately 100 and 560 mg/L, respectively, was evenly sprayed onto 30 kg of sterilized soil. The treated soil was thoroughly homogenized and aged for 30 d to ensure uniform contamination. The resulting Cd and Pb concentrations in the contaminated soil were 72.67 and 315.16 mg/kg, respectively. Details of the physicochemical properties of the initial and contaminated soils are provided in Table S3. The microbial inoculum was prepared according to protocols previously established by our research team (19).

### Pot experiment

The pot experiment was conducted from 17 May to 22 October 2022, in a greenhouse at the Baguazhou Experimental Base, Qixia District, Nanjing City, Jiangsu Province, China. During the experimental period, the ambient temperature ranged from 25.6°C to 35.1°C, relative humidity ranged from 60% to 75%, and photosynthetically active radiation (PAR) ranged from 14.50 to 17.15 MJ m^−2^ day^−1^ (recorded from 06:00 to 18:00). The photoperiod was maintained at 8 h ± 1 light and 16 h ± 1 dark.

The seeds of *A. fruticosa* L. were pretreated prior to transplantation. Initially, the seeds were soaked in distilled water for 12 h, followed by surface sterilization using 0.5% sodium hypochlorite solution for 1 min (20). After disinfection, seeds were rinsed thoroughly with sterile water and transferred to seedling trays for germination. Once the seedlings reached a uniform height of approximately 6–8 cm, they were transplanted into plastic pots (18 cm in diameter and 23 cm in height) filled with 2.5 kg of experimental soil. One healthy seedling was planted per pot. The experiment was structured into two soil conditions: uncontaminated initial soil and Cd- and Pb-contaminated soils. Eight treatments were established, each with three biological replicates. Each replicate consisted of one pot containing a single *A. fruticosa* L. seedling, resulting in a total of 24 pots (8 treatments × 3 replicates). The treatments were as follows: (i) CK, *A. fruticosa* L. in initial soil inoculated with sterile LB medium; (ii) JG1, *A. fruticosa* L. in initial soil inoculated with strain JG1; (iii) 2G5, *A. fruticosa* L. in initial soil inoculated with strain 2G5; (iv) 2G6, *A. fruticosa* L. in initial soil inoculated with strain 2G6; (v) HMCK, *A. fruticosa* L. in contaminated soil inoculated with sterile LB medium; (vi) HMJG1, *A. fruticosa* L. in contaminated soil inoculated with strain JG1; (vii) HM2G5, *A. fruticosa* L. in contaminated soil inoculated with strain 2G5; and (viii) HM2G6, *A. fruticosa* L. in contaminated soil inoculated with strain 2G6. One week after transplantation, 100 mL of microbial inoculum (OD_600_: 0.8–1.2, with the viable cell density of 0.8–1.2 × 10^8^ CFU/mL) was applied to the root zone of *A. fruticosa* L. seedlings in the designated treatments. This initial application marked day 1 of the pot experiments, and inoculation was repeated at monthly intervals. Between inoculations, 100 mL of sterile water was applied daily to maintain soil moisture. All pots were repositioned randomly each month to minimize positional effects.

### Plant analysis

After the completion of the pot experiment, plant samples were harvested. After oven-drying the plants at 60°C for 24 h until constant weight, the dry weights of roots, stems, and leaves were recorded using a precision electronic analytical balance to determine the total biomass. The concentrations of Cd and Pb in plant tissues were subsequently quantified. For HM determination, 0.1000 g of air-dried ground plant tissue (sieved to >100 mesh) was digested using concentrated sulfuric acid (H_2_SO_4_) and hydrogen peroxide (H_2_O_2_) in a 2:1 (vol/vol) ratio for 30 min. The digestate was cooled, transferred to a 50 mL volumetric flask, brought to volume with 2% nitric acid (HNO_3_), and filtered through a 0.45 μm membrane. The concentrations of Cd and Pb in the filtrate were determined using inductively coupled plasma mass spectrometry (ICP-MS). The concentrations of Cd and Pb in the whole plant and aerial parts were calculated using the following formulas. Among them, C_plant_, C_above_, C_root_, C_stem_, and C_leaf_ represent the concentrations of HMs in the whole plant, aerial parts, roots, stems, and leaves, respectively; and M_root_, m_stem_, and m_leaf_ represent the biomass of roots, stems, and leaves, respectively.


(1) $$C_{plant}(mg/kg)=\frac{m_{root}\times C_{root}+m_{stem}\times C_{stem}+m_{leaf}\times C_{leaf}}{m_{root}+m_{stem}+m_{leaf}}$$

(2) $$C_{above}(mg/kg)=\frac{m_{stem}\times C_{stem}+m_{leaf}\times C_{leaf}}{m_{stem}+m_{leaf}}$$

### Soil analysis

Rhizosphere soil samples were collected after the experiment. Soil attached to the root surface was obtained by manual shaking (approximately 60 times/min for 1 min). Fine roots were removed using tweezers to minimize interference during analysis. To determine total cadmium and lead (T_Cd, T_Pb), 0.1000 g of air-dried soil (sieved to >100 mesh) was digested with a mixed acid solution comprising nitric acid (HNO_3_), hydrofluoric acid (HF), and perchloric acid (HClO_4_) in a 6:2:1 (vol/vol/vol) ratio. After cooling, the digestate was transferred to a 50 mL volumetric flask, diluted with 2% HNO_3_ to a constant volume, and filtered. The concentrations of total Cd and Pb in the filtrate were quantified by ICP-MS. The available cadmium and lead (Y_Cd, Y_Pb) contents were extracted using diethylenetriamine pentaacetic acid (DTPA)-CaCl_2_-triethanolamine (TEA) solution, and the concentrations in the extracts were also analyzed by ICP-MS (21). The soil nutrient contents, including total nitrogen (TN), available nitrogen (AN), total phosphorus (TP), and available phosphorus (AP), were determined using the alkaline hydrolysis diffusion method and molybdenum-antimony anti-colorimetric method, respectively (22). Soil enzyme activities, including urease, protease, alkaline phosphatase, and sucrase activities, were also assessed. The urease activity was measured based on the release of ammonium (23). Protease activity was analyzed using casein as the substrate (24). Phosphatase activity was determined using disodium p-nitrophenyl phosphate (25). Sucrase activity was measured by 3,5-dinitrosalicylic acid colorimetry (26).

### Soil DNA extraction, PCR amplification, and high-throughput gene sequencing

Genomic DNA was extracted from the rhizosphere soil using an E.Z.N.A. Soil DNA Kit (Omega Bio-Tek, Norcross, GA, USA) according to the manufacturer’s instructions. DNA concentration and purity were assessed using Nanodrop RND-2000 (NanoDrop Technologies, USA) and 1% agarose gel electrophoresis. The V3–V4 hypervariable region of the bacterial 16S rRNA gene was amplified using primers 338F (5′-ACTCCTACGGGAGCAGCAG-3′) and 806R (5′-GGACTACHGGGTWTCTAAT-3′). The PCR mixture containing 2 µL of DNA (5–20 ng), 1 µL of each primer (10 µM), 12.5 µL of 2× Premix Taq (TaKaRa Bio Inc., Shiga, Japan), and 9 µL of ddH_2_O was subjected to thermal cycling to obtain the final amplified product. The PCR products obtained were pooled, purified using 2% agarose gel electrophoresis, and recovered using the AxyPrep DNA Gel Extraction Kit (Axygen Biosciences, USA). Quantification was performed using the QuantiFluor-ST system (Promega, USA). Raw sequencing data were filtered, and chimeric sequences were removed to generate optimized reads, which were clustered into operational taxonomic units (OTUs) based on 97% sequence similarity. High-throughput sequencing of purified amplicons was performed on the Illumina MiSeq platform (Illumina, San Diego, CA, United States) by Guangzhou Jidi’ao Technology Service Co., Ltd. (Guangzhou, China). The resulting raw sequencing reads were subjected to a standard bioinformatics pipeline. Briefly, sequences were quality-filtered, trimmed, and chimeras were removed. The remaining high-quality sequences were clustered into OTUs at a 97% similarity threshold. Subsequent analyses included the calculation of alpha diversity indices (Chao1, ACE, Shannon, Simpson), the assessment of beta-diversity using ordination methods, the examination of microbial community composition at different taxonomic levels, and the construction of co-occurrence networks to infer microbial interactions. Detailed procedures are provided in the Supplementary Material.

### Bioinformatics analysis and statistical analysis

**Fig 1:**
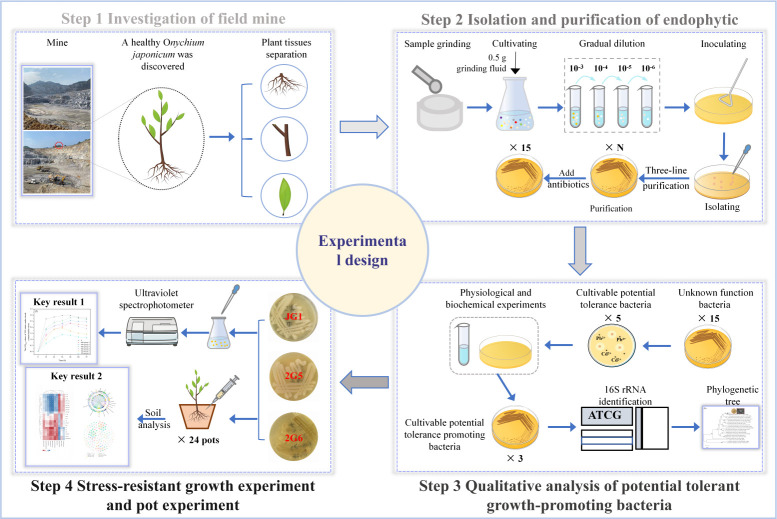
A flow diagram of mine investigation (plant sampling), microbial screening, functional evaluation of tolerance-promoting bacteria, and pot experiment. The photograph of the sampling site is republished from reference 27 (previously published under a CC BY license), reflecting the common origin of the research material.

All measured indicators were statistically analyzed using SPSS software (version 26.0, IBM, USA) through ANOVA to assess treatment effects. Prior to ANOVA, the normality and homogeneity of variance were tested using the Shapiro–Wilk test. The null hypothesis (H_0_) assumed no significant differences among treatments, whereas the alternative hypothesis (H_1_) posited that at least one treatment group differed significantly. Statistical significance was set at *P* < 0.05. Graphical representations were generated using Origin software (version 2024, Origin Lab, Northampton, Massachusetts, USA). For the analysis of rhizosphere bacterial communities, the processed OTU table (obtained as described in “Soil DNA extraction, PCR amplification, and high-throughput gene sequencing,” above) was used. Rhizosphere soil microbial diversity was evaluated based on OTU data. Diversity indices, including Chao1, ACE, Shannon, and Simpson, were calculated using the “Vegan” R package (https://cran.r-project.org/). RDA was performed using Canoco software (version 4.5) to assess correlations among soil environmental parameters, sample compositions, and microbial communities. Prior to RDA, all environmental variables were standardized using the default R base package to ensure data consistency. Spearman correlation analysis (nonparametric) was used to assess the relationships between environmental variables (e.g., soil HMs, nutrients, and enzyme activities) and bacterial communities. The microbial network structure was visualized using the “iGraph” R package. The variation partitioning analysis (VPA) was conducted to quantify the relative contributions of environmental factors to microbial community composition, with visualization performed via the “Vegan” R package. Partial least squares path modeling (PLS-PM) was conducted using the “plspm” package in R (v 4.3.0) to evaluate the path coefficients of latent variables (e.g., plant biomass, plant extraction efficiency, soil nutrients, enzyme activity, and microbial diversity) affecting soil Cd and Pb levels (Fig. 1).

## RESULTS

### Stress-resistant growth experiment to quantitatively evaluate the adaptability of resistant bacteria

**Fig 2:**
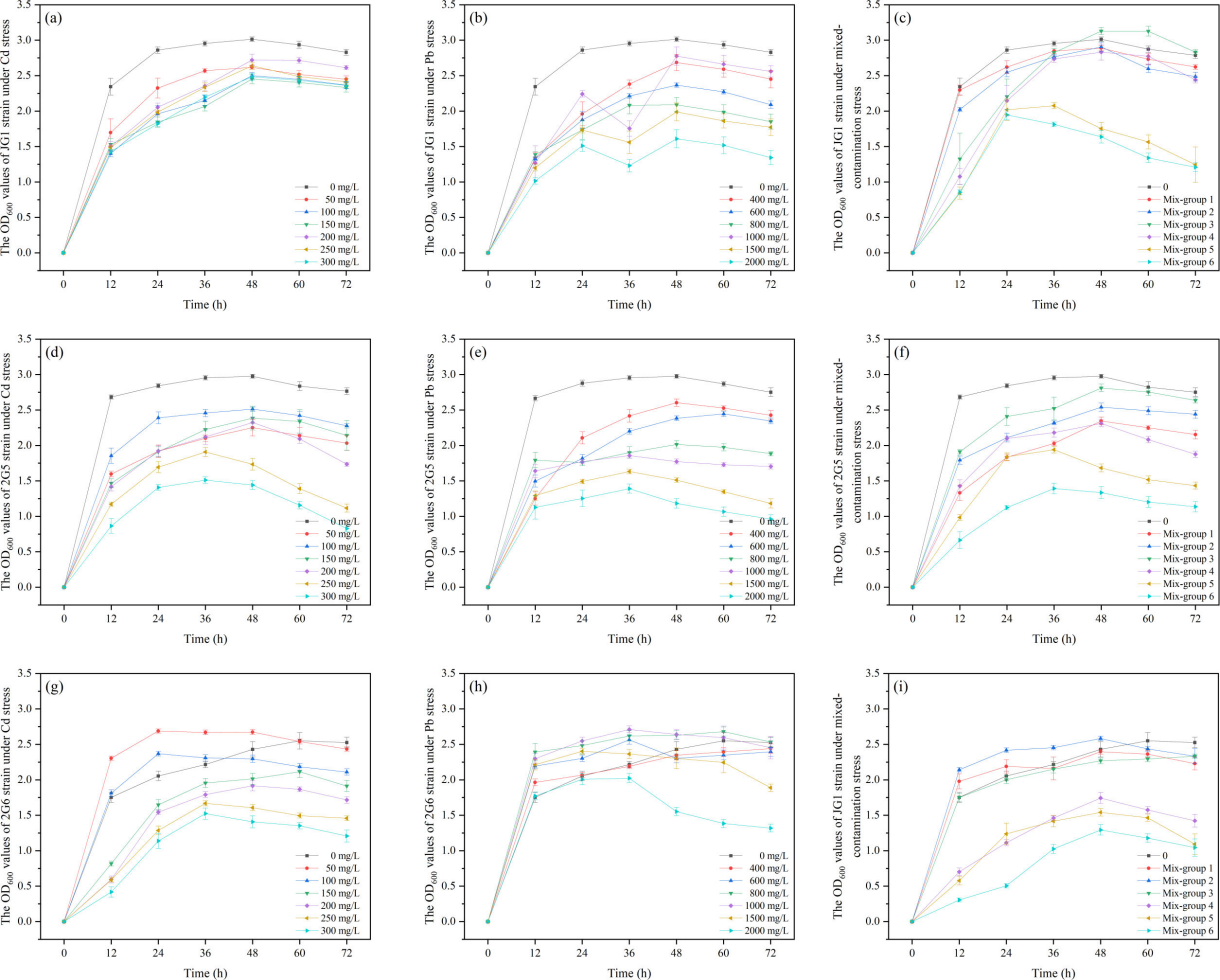
Growth of tolerance-promoting strains (JG1, 2G5, and 2G6) in culture solutions with different HM treatments: Cd²^+^ only (**a, d, g**), Pb²^+^ only (**b, e, h**), and Cd²^+^ + Pb²^+^ mixed (**c, f, i**).

The results demonstrated that the three tolerance-promoting bacterial strains (JG1, 2G5, and 2G6) exhibited distinct growth patterns at various concentrations of Cd^2+^, Pb^2+^, and their mixtures (Fig. 2). In Cd^2+^ medium, JG1 displayed the most robust growth, with peak absorbance values exceeding 2.0 CFU/mL across all tested concentrations (Fig. 2a). The absorbance values of 2G5 and 2G6 also exceeded 2.0 CFU/mL but only at Cd^2+^ concentrations below 200 and 100 mg/L, respectively. In Pb^2+^ medium, JG1, 2G5, and 2G6 reached absorbance values above 2.0 CFU/mL at Pb^2+^ concentrations of 600, 600, and 1,000 mg/L, respectively. In the mixed HM medium (Cd^2+^ and Pb^2+^), JG1 maintained growth above the 2.0 CFU/mL threshold up to Mix-group 4 (Cd^2+^: 200 mg/L; Pb^2+^: 1,000 mg/L), whereas 2G5 and 2G6 sustained such growth only up to Mix-group 3 (Cd^2+^: 200 mg/L; Pb^2+^: 600 mg/L) (Fig. 2c). These findings indicated that JG1, 2G5, and 2G6 demonstrated considerable tolerance and adaptability under both single and combined HM stress.

Considering the Cd (72.67 mg/kg) and Pb (315.16 mg/kg) levels in the contaminated soil used for the pot experiment (Table S1), these strains were deemed suitable for subsequent phytoremediation trials.

### Effects of inoculation with tolerance-promoting bacteria on biomass and accumulation of Cd and Pb in *A. fruticosa* L

**Fig 3:**
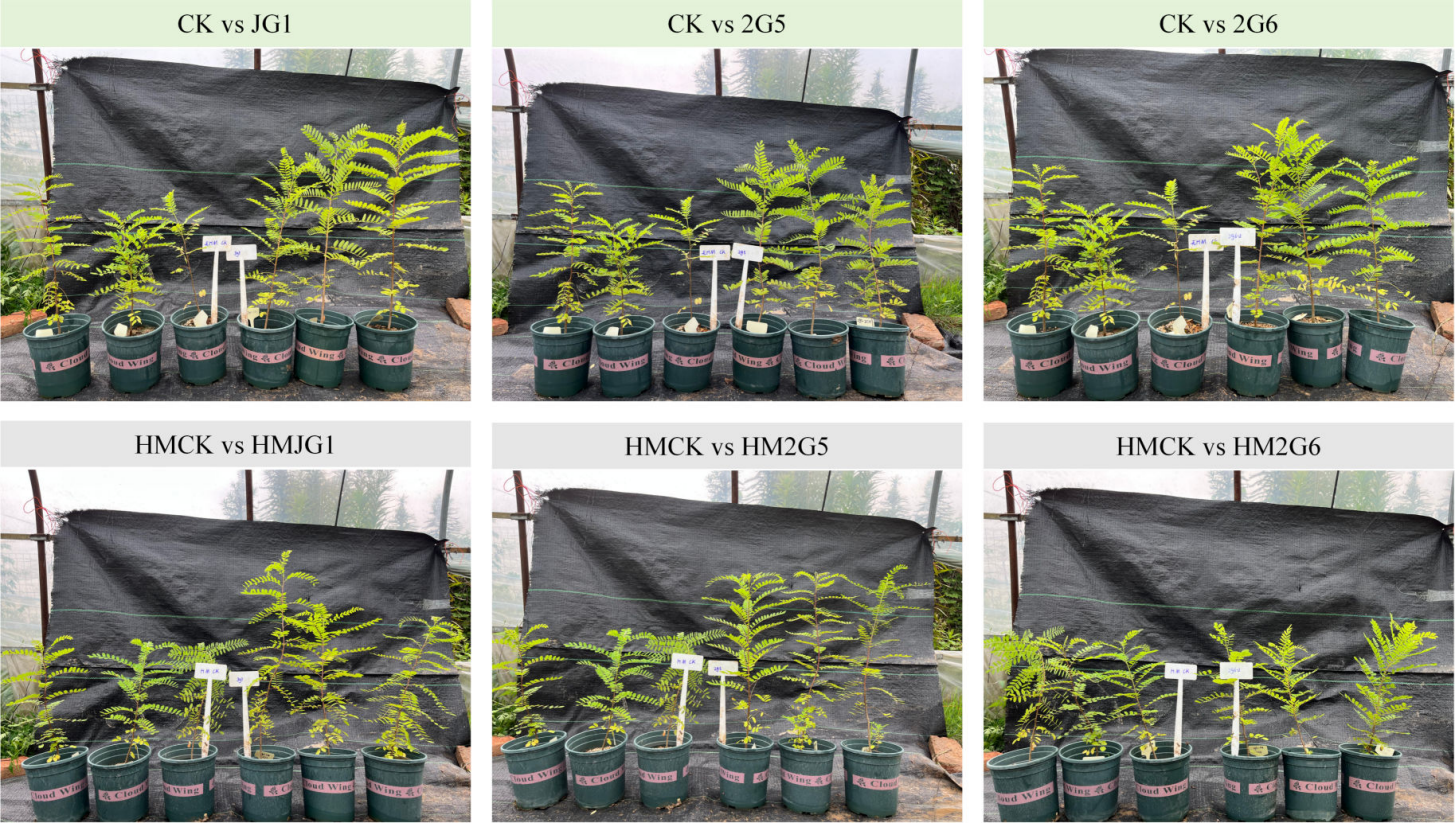
Growth of *A. fruticosa* L. in initial soil vs. Cd/Pb-contaminated soil with/without tolerance-promoting strain inoculation (JG1, 2G5, and 2G6).

*A. fruticosa* L. exhibited robust growth in both initial and Cd/Pb-contaminated soils (Fig. 3), and inoculation with tolerance-promoting bacteria further enhanced its biomass (Fig. S3). Compared with the control groups (CK and HMCK), the total biomass, aboveground biomass, and root biomass of *A. fruticosa* L. in the inoculation treatments (JG1, 2G5, and 2G6) increased by 13.71%–32.20%, 12.68%–57.18%, and 11.16%–20.63%, respectively. Notably, the biomass-promoting efficacy of the strains differed between soil conditions: the order was JG1 > 2G5 > 2G6 in uncontaminated soil, but 2G5 > JG1 > 2G6 in contaminated soil.

**Fig 4:**
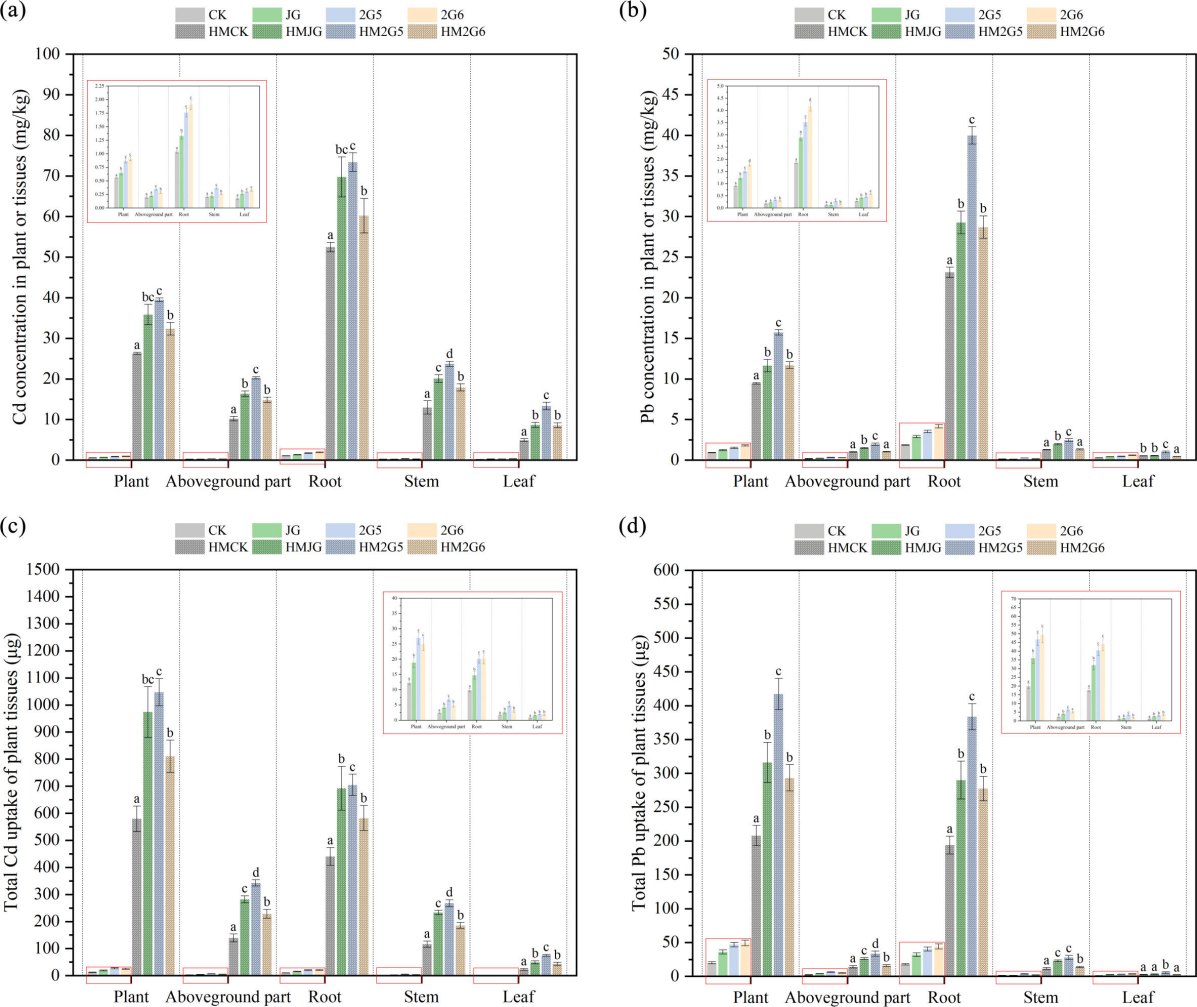
Effect of application of microbial inoculations on the concentrations of Cd (**a**) and Pb (**b**), and the uptake content of Cd (**c**) and Pb (**d**) of *A. fruticosa* L. in initial and contaminated soils. Different lowercase letters over bars indicate significant differences among different treatments (*P* < 0.05, *n* = 3, Tukey’s test).

As shown in Fig. 4a and b, under identical soil conditions, the concentrations of Cd and Pb in *A. fruticosa* L. tissues were significantly increased by bacterial inoculation. Cd and Pb accumulated predominantly in the roots, with notably lower concentrations in stems and leaves (<2.5 mg/kg). Compared with the HMCK, the total Cd concentration (All-Cd) in *A. fruticosa* L. was increased under the inoculation treatments. HM2G5 was the most effective, increasing All-Cd from 26.31 to 39.49 mg/kg. In particular, HM2G5 increased the Cd concentrations in root, stem, and leaf tissues from 52.49 mg/kg, 12.94 mg/kg, and 4.92 mg/kg to 73.40 mg/kg, 23.72 mg/kg, and 13.32 mg/kg, respectively. The Pb accumulation exhibited similar trends. Compared with HMCK, the total Pb concentration (All-Pb) was increased under inoculation treatments. HM2G5 again proved most effective, raising the root Pb concentration from 23.13 to 39.99 mg/kg. Although increases in Pb levels were also observed in stems and leaves, their overall concentrations were considerably lower than those in roots and were thus considered negligible.

Figure 4c and d illustrates that the total Cd and Pb accumulation in *A. fruticosa* L. was significantly enhanced by bacterial inoculation. The HM2G5 treatment resulted in the highest Cd accumulation in roots, stems, and leaves, reaching 704.44 μg/plant, 267.66 μg/plant, and 74.89 μg/plant, respectively. Regardless of treatment, Cd and Pb were predominantly accumulated in the roots, with the Pb content in roots reaching 383.71 μg/plant and accounting for over 90% of total plant accumulation. Furthermore, compared with HMCK (194.01 μg/plant), root Pb accumulation under HMJG1 and HM2G6 reached 290.04 and 277.35 μg/plant, respectively. Overall, bacterial inoculation markedly enhanced the Cd and Pb accumulation capacity of *A. fruticosa* L., with the accumulation efficiency ranking as follows: 2G5 > JG1 > 2G6.

### Effects of inoculation with tolerance-promoting bacteria on rhizosphere soil properties of *A. fruticosa* L

As shown in Table 2, the inoculation treatments affected the rhizosphere soil properties of *A. fruticosa* L. in both the initial and the contaminated soils. Compared with the initial soil, Cd and Pb contamination decreased soil nutrient availability and enzyme activity, an effect that was mitigated by bacterial inoculation. In contaminated soil, compared with HMCK, HMJG1 and HM2G5 both significantly increased the content of AN (HMJG1: 90.56 mg/kg; HM2G5: 100.11 mg/kg) and AP (HMJG1: 100.11 mg/kg; HM2G5: 96.76 mg/kg). Soil enzyme activities (urease, phosphatase, catalase, and sucrase) were significantly increased by inoculation in both soil types, except for a decrease in sucrase activity under the 2G6 treatment in uncontaminated soil. Notably, in contaminated soil, HM2G5 most effectively increased urease, phosphatase, and sucrase activities by 12.1%, 47.3%, and 44.4%, respectively, whereas HMJG1 had the greatest promoting effect on catalase activity, increasing it by 63.38%. Furthermore, inoculation treatments decreased the content of total Cd (T_Cd) and Pb (T_Pb) in contaminated soil compared to HMCK. Among them, HMJG1 treatment was particularly effective, decreasing T_Cd and T_Pb from 65.96 to 50.78 mg/kg and from 304.73 to 243.11 mg/kg, respectively. Notably, the effects of inoculation treatments on the available metal forms of Cd/Pb were different (Table 2). Specifically, compared with the HMCK, HMJG1 significantly decreased the content of Y_Cd and Y_Pb, HM2G6 significantly increased both, whereas HM2G5 treatment resulted in a reduction in Y_Cd and an increase in Y_Pb.

**TABLE 2:** Effects of microbial inoculation on HMs, enzyme activities, and nutrient content in rhizosphere soil of *A. fruticosa* L^*a*^

Treatment	CK	JG	2G5	2G6	HMCK	HMJG	HM2G5	HM2G6
T_Cd (mg/kg）	2.06 ± 0.11 c	1.64 ± 0.08 a	1.84 ± 0.08 b	1.72 ± 0.08 a	65.96 ± 2.50 e	50.78 ± 1.73 d	52.40 ± 4.10 d	53.79 ± 1.78 d
Y_Cd (mg/kg)	0.50 ± 0.04 b	0.45 ± 0.03 ab	0.41 ± 0.04 a	0.60 ± 0.03 c	20.70 ± 1.10 f	15.66 ± 1.74 e	14.96 ± 0.45 d	26.71 ± 0.56 g
T_Pb (mg/kg)	64.70 ± 1.01 d	50.12 ± 1.91 a	58.32 ± 0.80 c	56.12 ± 0.92 b	304.73 ± 9.43 g	243.11 ± 7.52 e	257.50 ± 3.82 f	246.54 ± 7.63 e
Y_Pb (mg/kg)	19.13 ± 1.38 a	23.45 ± 1.25 b	30.12 ± 2.23 c	22.11 ± 1.62 ab	80.37 ± 2.19 e	74.72 ± 5.30 d	135.92 ± 4.50 f	115.81 ± 4.57 g
TN (g/kg)	1.33 ± 0.01 d	1.28 ± 0.02 c	1.29 ± 0.03 cd	1.28 ± 0.03 c	1.22 ± 0.03 a	1.16 ± 0.02 a	1.18 ± 0.03 a	1.18 ± 0.03 a
TP (g/kg)	1.19 ± 0.03 b	1.27 ± 0.04 c	1.25 ± 0.06 c	1.26 ± 0.03 c	1.04 ± 0.02 a	1.18 ± 0.03 b	1.15 ± 0.06 b	1.06 ± 0.03 a
AN (mg/kg)	94.84 ± 4.52 b	120.09 ± 4.74 d	113.74 ± 7.19 c	105.99 ± 4.29 cd	74.21 ± 3.09 a	90.56 ± 1.87 b	100.11 ± 4.77 bc	105.99 ± 4.37 cd
AP (mg/kg)	124.46 ± 4.02 d	135.11 ± 8.75 e	138.55 ± 7.70 e	102.78 ± 7.30 c	84.08 ± 4.01 a	95.90 ± 1.60 b	96.76 ± 2.82 b	83.48 ± 1.36 a
Urease (U/g)	200.65 ± 1.54 d	220.61 ± 2.60 f	233.79 ± 2.36 g	210.46 ± 3.94 e	164.50 ± 2.21 a	176.10 ± 3.83 bc	184.46 ± 6.90 c	173.80 ± 2.83 b
Phosphatase (U/g)	10.43 ± 0.38 d	11.95 ± 0.26 e	13.36 ± 0.60 f	11.53 ± 0.47 e	6.56 ± 0.53 a	8.05 ± 0.18 b	9.66 ± 0.13 c	7.32 ± 0.29 a
Catalase (U/g)	12.96 ± 0.61 c	14.86 ± 0.73 d	14.29 ± 0.56 d	13.28 ± 0.68 c	7.10 ± 0.28 a	11.60 ± 0.79 c	9.19 ± 0.51 b	8.61 ± 0.46 b
Sucrase (U/g)	2.33 ± 0.19 c	2.62 ± 0.12 cd	2.89 ± 0.12 d	2.22 ± 0.21 c	1.71 ± 0.13 a	1.96 ± 0.16 b	2.47 ± 0.09 c	1.87 ± 0.14 ab

^*a*^ Different lowercase letters indicate significant differences between different microbial inoculations under identical soil conditions (*P* < 0.05, *n* = 3, Tukey’s test). TN, AN, TP, and AP represent total nitrogen, available nitrogen, total phosphorus, and available phosphorus, respectively. T_Cd and T_Pb represent total amount of Cd and Pb, respectively. Y_Cd and Y_Pb represent available amount of Cd and Pb, respectively.

### Effects of inoculation with tolerance-promoting bacteria on microbial diversity in rhizosphere soil of *A. fruticosa* L

The rhizosphere soil bacterial communities from 24 samples were analyzed using high-throughput Illumina MiSeq sequencing, yielding a total of 1,998,180 bacterial sequences across all samples, clustered into 58,143 OTUs, with an average sequencing coverage rate of 99.27%. The OTU dilution curve of the samples was flat, confirming the high quality and reliability of the sequencing data (Fig. S4).

**Fig 5:**
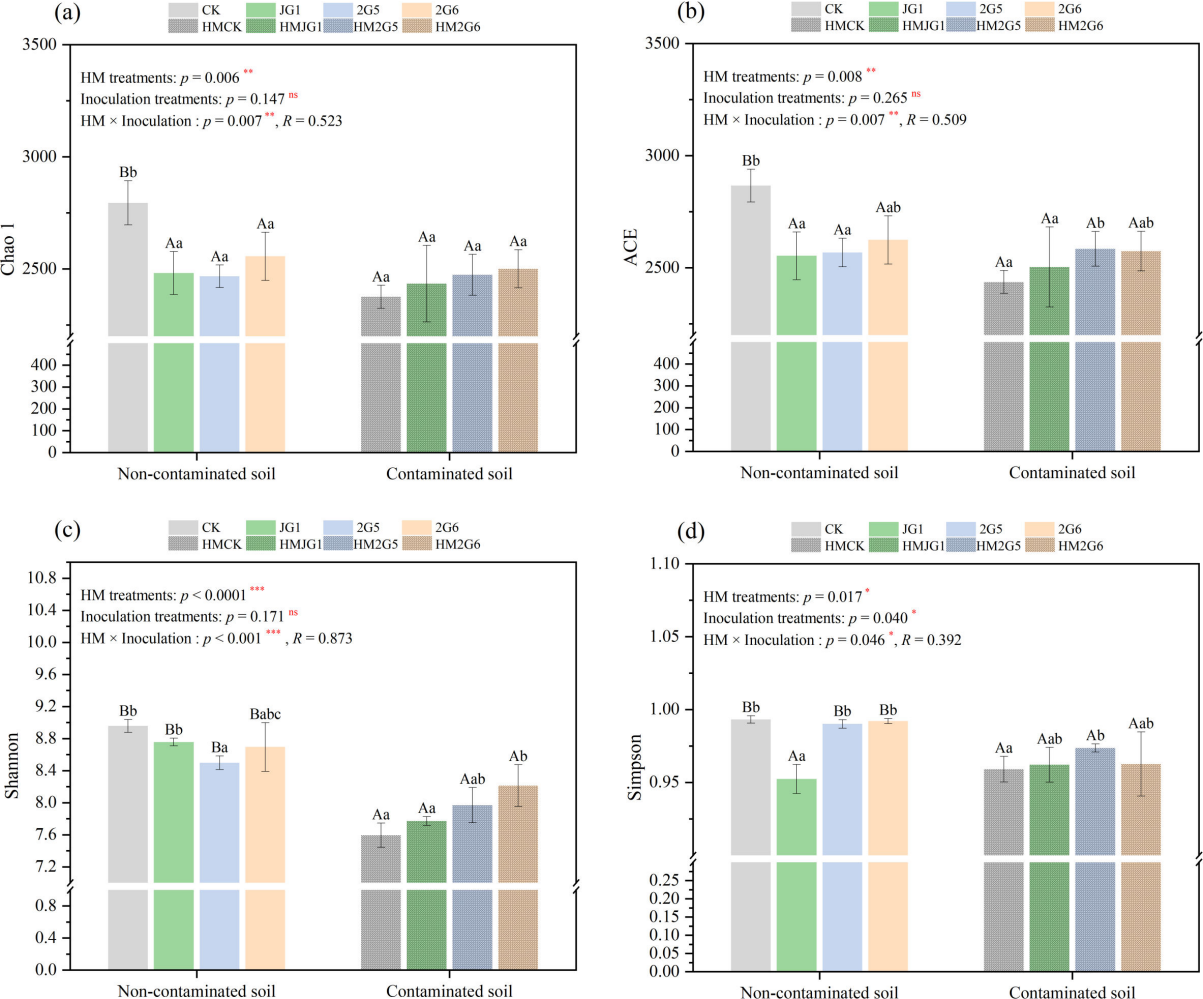
Effects of microbial inoculations on alpha diversity indices in rhizosphere soil of *A. fruticosa* L. Chao 1 (**a**), ACE (**b**), Shannon (**c**), and Simpson (**d**) indexes. *, **, and *** denote significant differences between different treatments at *P* < 0.05, *P* < 0.01, and *P* < 0.001, respectively; ns denotes no significance. Different lowercase letters indicate significant differences between different microbial inoculations under identical soil conditions. Different capital letters indicate significant differences between different soils under the same inoculation conditions.

Bacterial alpha diversity was evaluated using Chao1, ACE, Shannon, and Simpson indices (Fig. 5). As shown in Fig. 5, HM contamination significantly influenced Chao1 (*P* = 0.006), ACE (*P* = 0.008), Shannon (*P* < 0.001), and Simpson (*P* = 0.017) indices. In contrast, inoculation treatments had no significant effect on any of these indices under the initial soil. Compared with CK, JG1 significantly reduced Chao1, ACE, and Shannon indices; 2G5 also decreased Chao1, ACE, and Shannon indices, while 2G6 had no notable effect on any alpha diversity metrics. In contaminated soil, all diversity indices were significantly lower than those in uncontaminated soil under the same inoculation treatment, whereas inoculation effectively increased the indices compared with HMCK. Specifically, HM2G5 significantly increased ACE and Simpson indices, and HM2G6 significantly decreased the Simpson index, whereas other treatments showed no significant changes. These results demonstrated that microbial inoculation altered soil bacterial richness and diversity in a manner dependent on the contamination status of the soil.

### Effects of inoculation with tolerance-promoting bacteria on the structure and composition of rhizosphere soil microbial community of *A. fruticosa* L

The NMDS analysis indicated that both HM contamination and microbial inoculation induced varying degrees of deviation in the clustering patterns of rhizosphere soil bacterial communities (stress = 0.091, Fig. S5). Among the treatments, the bacterial community structure in soil inoculated with 2G5 under the same soil conditions exhibited the most pronounced deviation from the control groups. These findings suggested that both HM contamination and microbial inoculants significantly altered the soil bacterial community, with the strongest effects observed in the 2G5-inoculated group.

**Fig 6:**
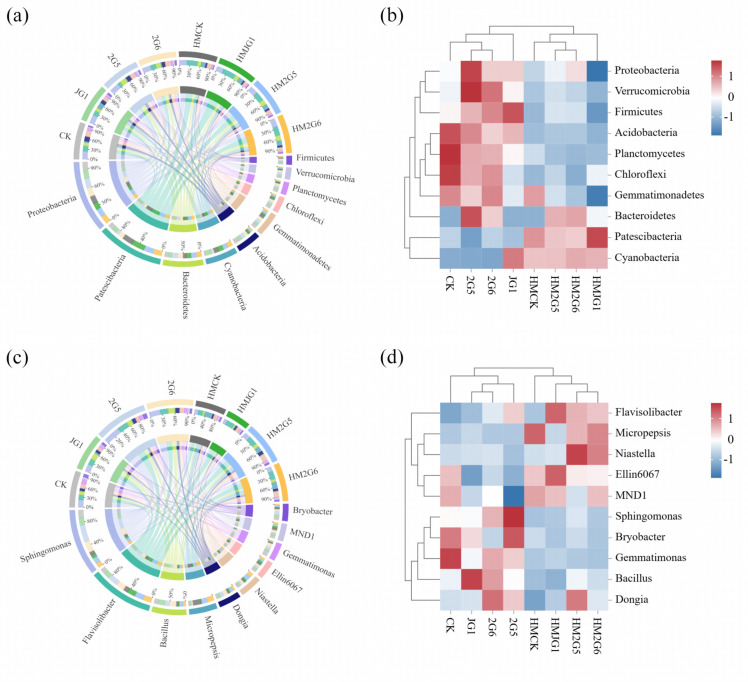
Effects of microbial inoculation on relative abundance (**a, c**) and heatmap (**b, d**) distribution of rhizosphere soil bacterial communities in *A. fruticosa* L. at phylum (**a, b**) and genus (**c, d**) levels. The color scale on the right indicates *R* values.

The effects of all treatments on the 10 most abundant bacterial phyla in the rhizosphere of *A. fruticosa* L. were further examined (Fig. S6). Among these taxa, all but *Bacteroidetes* were significantly affected by HM contamination. The inoculation treatments significantly influenced the relative abundances of *Patescibacteria*, *Bacteroidetes*, *Gemmatimonadetes*, *Chloroflexi*, *Planctomycetes*, and *Verrucomicrobia*. Moreover, the relative abundances of *Bacteroidetes*, *Gemmatimonadetes*, and *Planctomycetes* were affected by both HM contamination and microbial inoculation. Inoculation treatments decreased the relative abundance of *Gemmatimonadetes* in both initial and contaminated soils by 11.12%–25.10% and 24.94%–50.76%, respectively, whereas that of *Planctomycetes* decreased by 28.00% in contaminated soils. Notably, *Proteobacteria* dominated the initial soil, but *Patescibacteria* became the most abundant phylum under HM contamination, exceeding *Proteobacteria* in all inoculated treatments. Specifically, in contaminated soils, compared with HMCK, HMJG1 significantly decreased the relative abundance of *Proteobacteria* from 19.95% to 17.49%, and increased that of *Patescibacteria* from 27.51% to 34.57%. Furthermore, cluster analysis of the heat map revealed that these species in soils inoculated with 2G5 and 2G6 exhibited high similarity, whereas these species in HMJG1 differed most significantly from the control group (Fig. 6d).

At the genus level (Fig. S7), HM contamination significantly influenced the abundances of *Sphingomonas*, *Bacillus*, *Micropepsis*, *Dongia*, *Gemmatimonas*, and *Bryobacter*. The inoculation treatments exerted a significant effect on *Sphingomonas*, *Flavisolibacter*, *Bacillus*, *Micropepsis*, and *Dongia*. The interaction of HM contamination and microbial inoculation significantly affected *Sphingomonas*, *Bacillus*, *Micropepsis*, and *Gemmatimonas*. Among them, *Sphingomonas* was the dominant genus in the initial soil, while the inoculation treatments under HM contamination shifted the dominant genus to *Flavisolibacter*, which replaced *Sphingomonas*. Specifically, compared with CK, 2G5 treatment significantly increased the relative abundance of *Sphingomonas* from 3.20% to 7.22%, and *Flavisolibacter* from 2.08% to 4.77%. Similarly, compared with HMCK, HM2G5 treatment increased *Sphingomonas* from 1.55% to 2.55%, and *Flavisolibacter* from 2.40% to 3.82%. Additionally, HM2G5 significantly increased the relative abundance of *Niastella* (from 0.75% to 2.17%), *Micropepsis* (from 0.66% to 2.26%), and *Bacillus* (from 0.77% to 1.50%), whereas HM2G6 only significantly increased the relative abundance of *Micropepsis* and *Bacillus*. In contrast, HMJG1 had no significant effect on these genera. Thermal map clustering (Fig. 6d) revealed that the influence of inoculation treatments on these species varied between the initial soil and the contaminated soil. In the initial soil, the bacterial community in CK was most similar to that in 2G5. However, in contaminated soil, HMJG1 and HMCK treatments exhibited the highest similarity.

**Fig 7:**
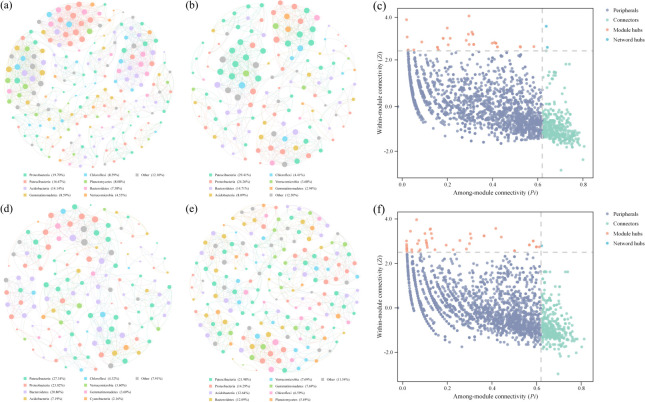
Co-occurrence networks of soil bacterial community (**a, b, d, and e**), and the *Zi-Pi* plots (**c and f**) of the distribution of core microbiomes based on their topological roles in networks under different treatments. (**a**) CK treatment, (**b**) TRE treatment: microbial inoculation treatments (including JG1, 2G5, and 2G6 treatments), (**d**) HMCK treatments, and (**e**) HMTRE treatment: microbial inoculation treatments (including HMJG1, HM2G5, and HM2G6 treatments). The red or gray lines indicate positive or negative correlations. The dot size is positively correlated with the OTU abundance, and the dot color distinguishes the OTU at the phylum level. The threshold values of *Zi* and *Pi* for categorizing genus were 2.5 and 0.62, respectively.

Spearman correlation analysis (|r| > 0.6, *P* < 0.05) was employed to construct microbial symbiotic networks (Fig. 7). The network topology parameters are listed in Table S4. Compared with the control groups, the number of nodes, edges, network density, average degree, and average weighted degree were all reduced after inoculation, indicating weakened interspecific associations due to microbial agent inoculation. Despite this, the network modularity was enhanced: modularity under TRE treatment (including JG1, 2G5, and 2G6 treatments) increased from 1.129 to 2.407 and under HMTRE treatment (including HMJG1, HM2G5, and HM2G6 treatments) from 1.814 to 5.905. Additionally, inoculation treatments increased the proportion of positive edges and reduced that of negative edges. Regardless of inoculation treatments, positive correlations predominated over negative correlations. The proportion of positive to negative edges increased markedly under TRE and HMTRE treatments, from 168.60% to 231.67% and 210.17% to 352.28%, respectively. Compared with the control groups, the increased positive correlations suggested that the bacterial inoculants enhanced the internal cohesion within soil bacterial communities, thereby improving environmental adaptability and contributing to the establishment of dominant species interaction networks.

### Multivariate analysis

**Fig 8:**
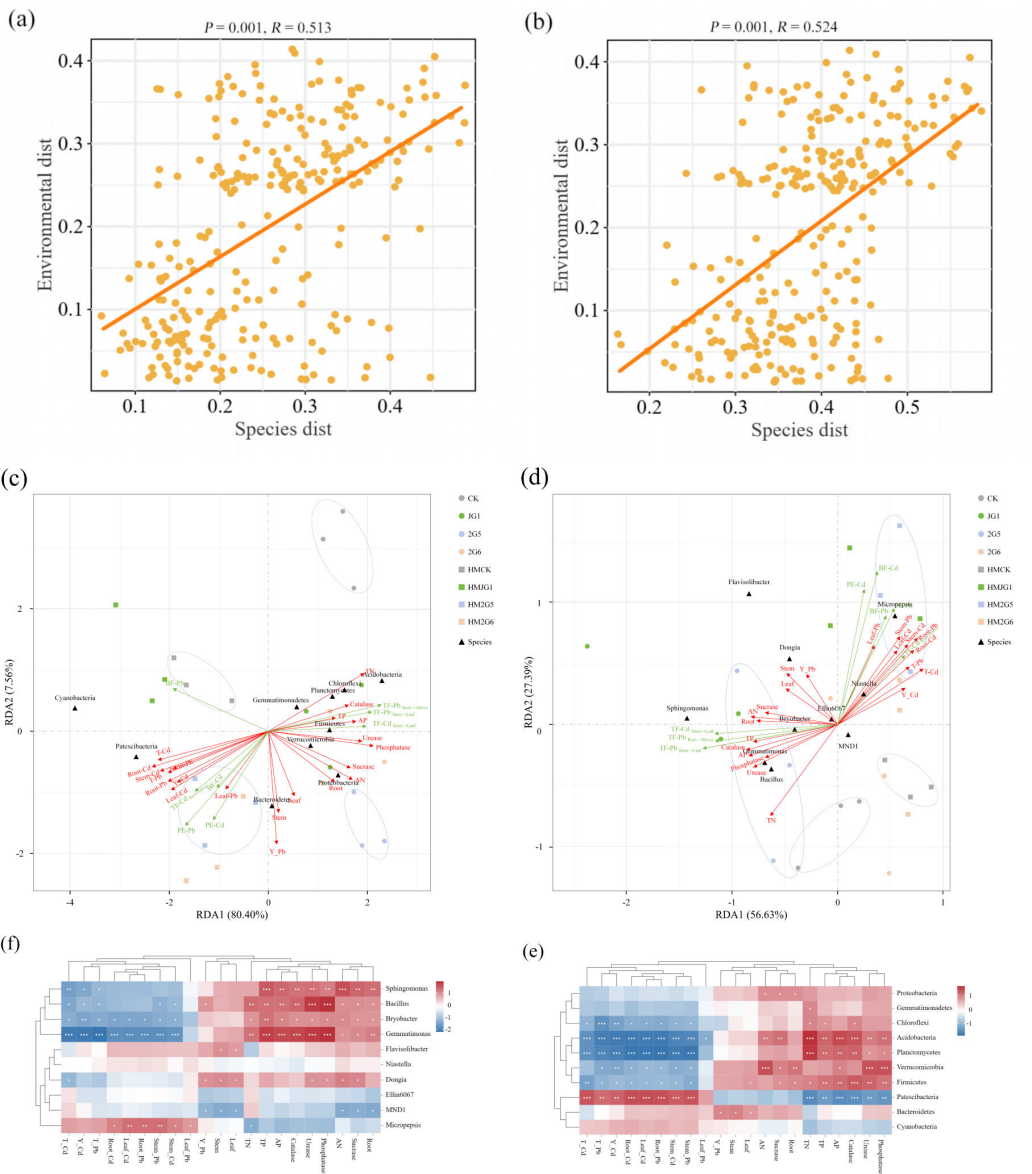
Linear relationships between environmental variables and bacterial communities at phylum (**a**) and genus (**b**) level based on Mantel test analysis. Redundancy analysis (RDA) of the environmental variables, soil samples, with phylum (**c**) and genus (**d**) level of soil bacterial communities. Heatmaps of Spearman correlations between bacterial composition at phylum (**e**) and genus (**f**) level with environmental variables. X- and Y-axes in panels c and d represent the two main coordinate axes, and the percentage values of the coordinate axes represent variance explained. The color scale on the right indicates *R* values. The asterisks denote significance levels: *, *P* < 0.05; **, *P* < 0.01; ***, *P* < 0.001. TN, AN, TP, and AP represent total nitrogen, available nitrogen, total phosphorus, and available phosphorus, respectively. T_Cd and T_Pb represent the total amount of Cd and Pb, respectively. Y_Cd and Y_Pb represent the available amount of Cd and Pb, respectively. Root, stem, and leaf represent the biomass of the root, stem, and leaf, respectively. Root_Cd, stem_Cd, and leaf_Cd represent the Cd concentration of the root, stem, and leaf, respectively. Root_Pb, stem_Pb, and leaf_Pb represent the Pb concentration of the root, stem, and leaf, respectively.

The Mantel test analysis indicated that all studied environmental factors significantly influenced the composition of the soil bacterial community at both the phylum (Fig. 8a) and genus levels (Fig. 8b). At the phylum level, the five most influential environmental factors were identified as T_Cd, TN, T_Pb, urease, and phosphatase, with the respective explanatory degrees of 28.56%, 27.19%, 27.13%, 26.43%, and 26.25% (Fig. S8). RDA further revealed that environmental variables explained 87.96% of the total variation in the bacterial community structure at the phylum level, with RDA1 and RDA2 accounting for 80.40% and 7.56% of the variation, respectively (Fig. 8c). Spearman correlation analysis (Fig. 8e) indicated that *Proteobacteria* was positively correlated with AN, sucrase activity, and root biomass. *Patescibacteria* showed positive correlations with T_Cd, T_Pb, Y_Cd, and plant HM concentrations and were negatively correlated with soil nutrient levels (except AN) and soil enzyme activities (excluding sucrase). In contrast, *Bacteroidetes* were positively associated with Y_Pb, stem biomass, and leaf biomass. At the genus level, the explanatory degrees of Phosphatase, Urease, T_Cd, Y_Pb, and TP were calculated to be 24.75%, 23.30%, 20.72%, 19.17%, and 19.09%, respectively, representing the five most influential environmental factors (Fig. S8). RDA at the genus level showed that environmental factors explained 84.02% of the variation in the bacterial community composition, with RDA1 and RDA2 accounting for 56.63% and 27.39%, respectively (Fig. 8c). Among individual genera, *Sphingomonas* was negatively correlated with HMs (except Y_Pb) and positively correlated with soil nutrients and enzyme activities. *Flavisolibacter* was positively associated with stem and leaf biomass. *Bacillus* exhibited negative correlations with soil HMs (except Y_Pb), Stem_Pb, and Stem_Cd, but was positively correlated with soil nutrient levels and soil enzyme activities.

**Fig 9:**
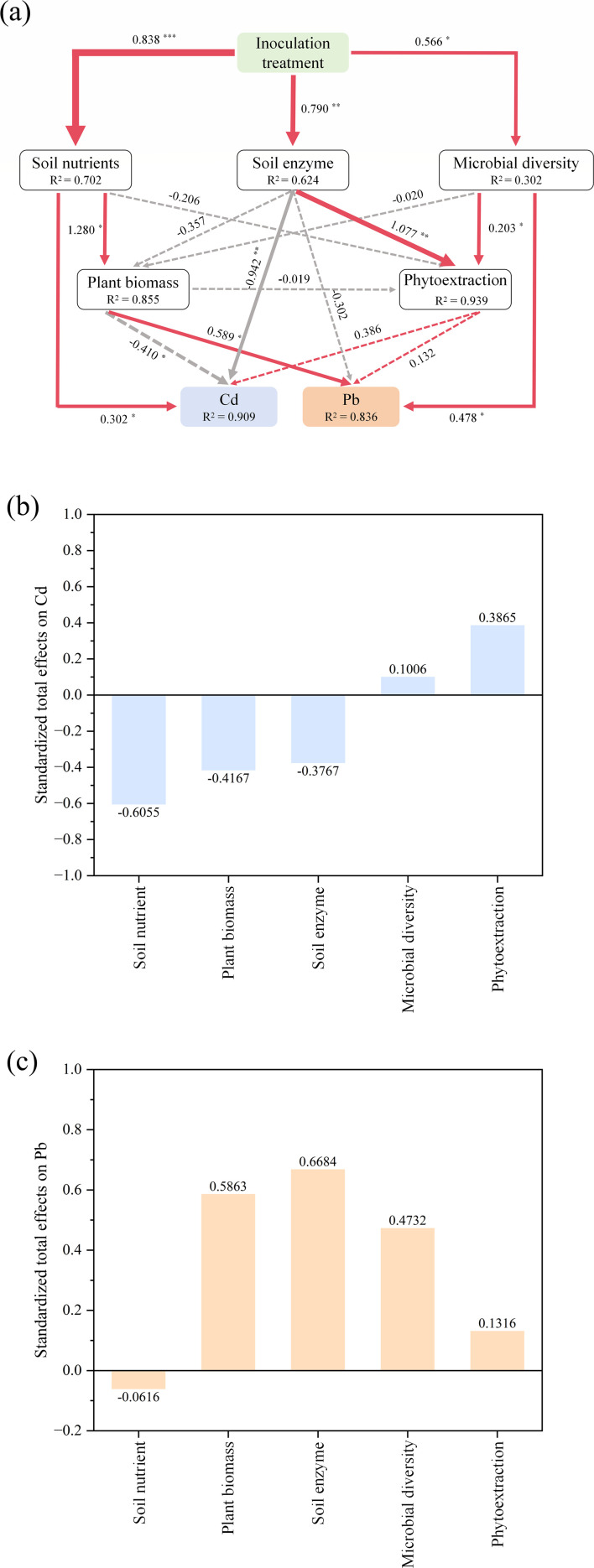
Partial least squares path model (PLS-PM, **a**) reveals the relationships between latent variables and soil Cd and Pb. (**b**) and (**c**) represent the normalized total effects of latent variables on Cd and Pb, respectively. The red line and the gray line represent the positive and negative correlations between latent variables, respectively. Solid and dashed arrows represent significant and non-significant relationships, respectively. The number of asterisks indicates the degree of correlation: *, *P* < 0.05; **, *P* < 0.01; ***, *P* < 0.001. The line width represents the correlation coefficient between latent variables, and the wider the line represents the greater the correlation coefficient value. The number above the line represents the path coefficients between latent variables.

Structural equation modeling (PLS-PM) was conducted to characterize the internal relationships among potential variables (including soil nutrients, soil enzyme activities, microbial diversity, plant biomass, and plant extraction efficiency) and soil Cd and Pb content after microbial inoculation (Fig. 9a). The inoculation treatments explained 90.9% and 83.6% of the total variation in soil Cd and Pb contents, respectively (Fig. 9a). The results demonstrated that the microbial inoculation significantly affected soil nutrients, enzyme activities, and microbial diversity, with the path coefficients of 0.838 (*P* < 0.001), 0.790 (*P* < 0.01), and 0.566 (*P* < 0.05), respectively. Notably, soil nutrients, plant biomass, and soil enzyme activities exerted positive direct effects on Cd, with corresponding path coefficients of 0.302, −0.410, and −0.942, respectively. Soil nutrients had a direct positive effect on plant biomass (path coefficient = 1.280), but no direct effect on plant extraction efficiency was detected. For Pb, the microbial diversity was the only variable with a direct significant effect, with a path coefficient of 0.478. The standardized total effect values showed that the contributions of plant biomass and extraction efficiency to Cd were −0.417 and 0.387, respectively, whereas the total effect values for Pb were 0.586 and 0.132, respectively (Fig. 9b and c).

## DISCUSSION

### Effects of changes in plant characteristics on remediation of HM-contaminated soil

Traditional plant growth-promoting bacteria (PGPB) have been shown to enhance plant growth (or biomass) under uncontaminated soil conditions. To mitigate the functional loss caused by stress responses in PGPB under HM (Cd and Pb) contamination, it is necessary to clarify the interaction mechanisms between microorganisms and plants in such environments. In this study, the adaptability of three PGPB strains (JG1, 2G5, and 2G6) to Cd and Pb stress was evaluated using stress resistance assays. The absorbance threshold of 2.0 CFU/mL was adopted as the criterion for microbial adaptability following standard protocols (18). Three PGPB strains (JG1, 2G5, and 2G6) exhibited robust tolerance to single and mixed Cd/Pb stress, with growth viability maintained under metal concentrations matching the actual contaminated soil conditions used in the pot experiment. This adaptability is a key prerequisite for their practical application in field remediation, as strains with poor stress tolerance often fail to colonize and function effectively in HM-polluted soils (28, 29). This finding aligns with reports on other metal-tolerant bacteria, such as *Serratia marcescens* CP-13 and *Achromobacter mucilens* (28, 30). Moreover, given the Cd and Pb concentrations in the potted soil used in this study (Table S3), these strains demonstrated robust adaptability to the actual metal concentrations measured, enabling them to persist in contaminated rhizospheres and exert growth-promoting effects.

Plant biomass and net extraction capacity can play essential roles in the *in situ* remediation of HM-contaminated soils (4, 6). Inoculation with tolerance-promoting bacteria (JG1, 2G5, and 2G6) under both initial and contaminated soil conditions significantly enhanced the biomass of *A. fruticosa* L., with 2G5 outperforming the other strains (Table S2). This improvement could be attributed to the increased secretion of IAA, enhanced nitrogen fixation, and greater phosphorus solubilization capacity, all of which may alleviate metal stress and promote biomass accumulation. The microbial secretion of iron carriers may activate soil oxidase enzymes, reduce environmental stress, and sustain vital physiological processes, such as chlorophyll synthesis and respiration, serving as the key factors contributing to improved plant adaptation and growth (31–33). Furthermore, the positive effect of inoculation on plant biomass may result from microbial detoxification mechanisms. Through interactions with host plants, key enzymes such as cytoplasmic reductases and components of the thioredoxin pathway contribute to the reduction of HM bioavailability, mitigating their toxic effects on plant cells (34, 35). Improved soil nutrient status can further enhance plant growth. In this study, inoculation significantly increased the AN and AP contents in the soil by 11.76%–42.82% and 8.56%–15.08%, respectively, indicating improved soil fertility and enhanced nutrient availability. These increases could be caused by nitrogen fixation and phosphorus solubilization functions of the inoculated strains. Moreover, enhanced soil enzyme activities (e.g., phosphatase and urease) can promote the transformation of insoluble soil nutrients into bioavailable forms, facilitating uptake by plants and thereby improving plant growth (27, 36). Consistent with these findings, Liao et al. (37) reported increased rhizospheric enzyme activity and biomass in *Lolium perenne* following inoculation with *Enterobacter hormaechei* strain X20. Similarly, Liu et al. (34) observed a positive correlation between plant biomass and available soil nutrients (AN and AP). These results are consistent with those of previous studies. The structural equation modeling (PLS-PM) analysis further validated (Fig. 9) that soil nutrients significantly influenced the biomass of *A. fruticosa* L. (path coefficient = 1.280**), supporting the conclusion that changes in soil nutrient content served as a direct driver of biomass variation in *A. fruticosa* L.

Additionally, the accumulation of Cd and Pb in plants is a critical factor influencing soil HM content. In this study, inoculation with the 2G5 strain notably enhanced the extraction efficiency of Cd and Pb from roots, stems, and leaves of *A. fruticosa* L., with Cd and Pb concentrations in roots significantly exceeding those in other organs. This pattern is linked to plant structural and physiological traits, as well as microbe-mediated improvements in metal bioavailability (27). Specifically, the inoculated strains promote the coupling of soil HMs with iron and secrete organic acids (e.g., citric acid), which increase the solubility and mobility of Cd and Pb, facilitating their uptake and translocation from roots to shoots (29, 38). Notably, HMJG1 treatment significantly decreased the contents of Y_Cd, Y_Pb, and significantly increased Cd and Pb accumulation in *A. fruticosa* L. This may result from the plant’s metal extraction rate exceeding the increase in metal bioavailability induced by microbes, ultimately leading to a lower effective metal content in the soil. This is an important finding for HM remediation. It demonstrates that JG1 inoculation can enhance HM extraction by *A. fruticosa* L. without concurrently elevating environmental risks associated with increased metal mobility in the soil. Furthermore, PLS-PM (Fig. 8) analysis identified soil enzyme activity as a direct determinant of plant extraction efficiency. This aligns with Nantapong et al. (39), whicho reported that high antioxidant enzyme activity in the soil can mitigate oxidative stress, preserve cell membrane integrity, and enhance plant defense against reactive oxygen species. Collectively, these physiological improvements enable plants to better withstand environmental stress and sustain the continuous uptake of HMs under adverse conditions.

Indeed, our results supported the existence of an internal relationship among rhizospheric soil nutrients, soil enzyme activities, and the growth and metal tolerance of *A. fruticosa* L. However, this study lacked sufficient data on the internal mechanisms governing nutrient absorption and HM transport, particularly regarding the role of endogenous hormones and their interactions with metal accumulation in plant tissues. This limitation may inhibit the comprehension of nutrient transformation and HM translocation in *A. fruticosa* L. tissues. Therefore, further studies are required to elucidate the nutrient enrichment and transport mechanisms in *A. fruticosa* L. inoculated with tolerance-promoting bacteria, thereby validating the proposed hypothesis and providing a theoretical foundation for future applications.

### Influence of soil microbial community change on remediation of HM-contaminated soil

Inoculation of exogenous microorganisms and changes in the soil environment (e.g., HM contamination) can alter the diversity and structure of host microbial communities (40, 41). Our results confirmed that HM contamination exerted a greater impact on bacterial community composition than microbial inoculation, which is consistent with previous findings that abiotic stress often overrides biotic factors in shaping soil microbial assemblages (11, 42). Notably, inoculation with tolerant growth-promoting bacteria in contaminated soil reduced α-diversity, whereas it increased α-diversity in the initial soil. This may be due to the short-term restriction of bacteria with weak tolerance under HM stress, while those with strong tolerance were retained, thereby influencing bacterial α-diversity (18). Studies have also shown that changes in α-diversity caused by exogenous microorganisms may depend on the stability of soil microecology (11). In poor soil conditions with limited nutrients, ecological niches may shift as competition between introduced and native microorganisms occurs. After stabilization, a new microbial balance is established, which increases the ability to resist external interference and maintains ecological functions (42).

Additionally, inoculation with tolerance-promoting bacteria alters the bacterial community structure in rhizosphere soil, thereby shifting the microbial niche. In this study, a notable shift was the replacement of *Proteobacteria* (dominant in initial soil) by *Patescibacteria* as the most abundant phylum in contaminated soil. This shift underscores the strong adaptability of *Patescibacteri*a to HM stress, which can be attributed to their key soil metabolic functions, including carbohydrate and vitamin metabolism, within a short timeframe, thereby gaining a competitive advantage in HM-contaminated soil (43). Furthermore, Zhang et al. (40) confirmed that resistance genes (e.g., P gene and L gene) in *Patescibacteria* were significantly upregulated in response to HM stress, with enhanced tolerance achieved through the replication and recombination for gene repair. Spearman correlation analysis (Fig. 8) demonstrated a positive association between Patescibacteria and the concentrations of HMs in the soil (excluding Y_Pb) and plant tissues. This finding aligns with previous reports.

At the genus level, HM contamination and microbial inoculation triggered a reorganization of the rhizosphere community, with *Sphingomonas*, *Flavisolibacter*, *Bacillus*, and *Micropepsis* emerging as key functional taxa. These genera are well documented for their adaptability to extreme environments and capacity to enhance phytoremediation (30, 44). A significant shift observed was the replacement of *Sphingomonas* (dominant in uncontaminated soil) by *Flavisolibacter* under HM stress. This transition likely stems from the competitive advantage of *Flavisolibacter* in utilizing phosphorus and secreting bioactive metabolites (18, 30). *Sphingomonas*, on the other hand, maintained positive correlations with soil nutrients and enzyme activities, indicating its role in nutrient cycling and soil quality improvement. *Bacillus*, a well-recognized plant growth-promoting genus and HM remediation biomarker, contributed to enhanced nitrogen cycling and nutrient metabolism, thereby alleviating metal stress and promoting plant growth (18, 30). Its abundance was positively correlated with soil nutrient availability and enzyme activities, corroborating its functional importance. Collectively, these genus-level dynamics demonstrate the functional complementarity within the microbial community, in which key taxa synergistically modulate HM bioavailability and enhance plant stress tolerance, thereby driving the soil HM remediation process (30, 44).

The microbial symbiotic network illustrates the structure and relationships of the rhizosphere microbial community. Compared with the initial soil, the microbial network under HM-contaminated soil contained more template blocks, likely reflecting the coping strategies and functional changes of soil microbes in response to environmental fluctuations (45). Previous studies have indicated that mild environmental stress can improve microbial interactions while reducing interspecies competition, thus enhancing microbial community stability (24). In this context, inoculation with tolerance-promoting bacteria can further increase template block complexity and reduce the average degree and density of microbial networks, indicating a stabilization effect. These beneficial microorganisms may promote bacterial community stability by reducing network complexity and richness. In this study, inoculation with tolerance-promoting bacteria contributed to a higher proportion of positive interactions (Table S4), suggesting that the microbial community stability in polluted soils depended on the key microbial interactions. Some studies have also indicated that shifts in microbial network stability may reflect long-term synergistic adaptation processes under HM pressure (24, 45). When exogenous microorganisms colonize soil, host microbes rapidly adapt to short-term environmental fluctuations through metabolic modulation. Over time, the intake of beneficial microbes facilitates the recovery of microbial network stability, confirming the functional role of microbial inoculation in enhancing HM remediation capacity.

To elucidate the distinctive mechanisms, the phytoremediation performance of the *A. fruticosa* L.-bacteria (JG1, 2G5, and 2G6) system was compared with two previously studied plant-microbe systems: *Leucaena leucocephala*-Trichoderma harzianum J2 and *Sophora xanthantha*/*Robinia pseudoacacia-Paenibacillus polymyxa* WZ14. All three systems share the overarching strategy of enhancing soil nutrient availability and modulating rhizosphere microbial communities. However, distinct regulatory mechanisms were identified for the *A. fruticosa* L.-bacteria system. In the current study, the phylum *Patescibacteria* and the genus *Flavisolibacter* were identified as core bioindicators responsive to HM stress. In contrast, the *T. harzianum* J2 system involved shifts in fungal phyla such as *Chytridiomycota*, *Mucoromycota*, and the protist phylum *Ciliophora*, whereas the *P. polymyxa* WZ14 system was characterized by changes in bacterial genera including *Sphingomonas*, *Flavisolibacter*, *Bacillus*, and *Streptomyces*. Second, PLS-PM analysis revealed a distinct regulatory network for the *A. fruticosa* L.-bacteria system. Specifically, soil enzyme activity and plant biomass emerged as the dominant factors driving Cd remediation, while microbial diversity was the primary driver for Pb remediation. In contrast, the *T. harzianum* J2 system identified nitrogen-related soil properties (e.g., total nitrogen, available nitrogen) as the prominent drivers, while the *P. polymyxa* WZ14 system emphasized the synergistic effects of soil nutrients and key bacterial genera. Third, the *A. fruticosa* L-tolerance-promoting bacteria system exhibited a more significant enhancement in microbial network stability (higher modularity and positive interaction ratios), which contributed to stronger resistance against HM stress compared to the two previous plant-microbe combinations. Collectively, these differences underscore the specificity of plant-microbe symbiotic interactions in response to HM contamination. The *A. fruticosa* L.-growth-promoting bacteria (JG1, 2G5, and 2G6) consortium offers a distinct microbial regulation pathway, thereby complementing and extending our previous mechanistic findings.

### Conclusion

In this study, three potential tolerant growth-promoting bacterial strains were identified based on physiological and biochemical assessments, as well as Cd and Pb tolerance evaluations. Their growth capacities under Cd and Pb stress were quantitatively assessed through stress-resistant growth experiments. These strains were found to mitigate the adverse effects of HM contamination (Cd and Pb) on *A. fruticosa* L. and promote the accumulation of HMs in plants to varying degrees, with strain 2G5 exhibiting the strongest performance. Furthermore, the inoculation of tolerance-promoting bacteria enhanced soil properties, including nutrient levels and enzyme activities, which improved plant tolerance to HM stress and promoted growth. Principal coordinate analysis revealed that HM contamination and bacterial inoculation significantly altered the composition of the rhizosphere soil bacterial community. These changes involved shifts in the ecological niches of dominant taxa and improvements in microbial stability, suggesting that such inoculation may serve as an effective strategy for addressing HM stress. Tolerance-promoting bacteria may also alleviate the impact of HM contamination on plants by recruiting specific beneficial taxa such as p_*Patescibactria* and g_*Flavisolibacter*. In summary, these findings provide valuable insights into enhancing plant adaptability and metal remediation capacity under HM stress through the inoculation of beneficial microbes. Such strategies promote cooperation between microbial inoculants and host plants to remediate contaminated soils. Future studies should focus on the practical implementation of tolerance-promoting bacterial inoculation in field-scale applications to develop efficient and reliable bioremediation technologies for heavily polluted environments.

## Data Availability

The 16S rDNA sequences are available in NCBI/ENA/DDBJ under the following accession numbers: for *Serratia marcescens* JG1, accession no. OQ626703; for *Serratia marcescens* 2G5, accession no. OQ626626; and for *Achromobacter mucilens* 2G6, accession no. OQ626695.

## SUPPLEMENTAL MATERIAL

Supplemental material aem.00068-26-s0001.pdf Tables S1 to S6; Fig. S1 to S8.

ASM does not own the copyrights to Supplemental Material that may be linked to, or accessed through, an article. The authors have granted ASM a non-exclusive, world-wide license to publish the Supplemental Material files. Please contact the corresponding author directly for reuse.
