# Supplementary material for: Inoculation with cadmium/lead-tolerant bacteria enhances phytoremediation of Amorpha fruticosa L. by shifting key taxa and improving microbial stability
Source: Appl Environ Microbiol. 2026 May 15;92(6):e00068-26. doi: 10.1128/aem.00068-26 (PMC13274456; doi:10.1128/aem.00068-26)
Supplement: Supplemental material — Tables S1 to S6; Fig. S1 to S8. [file aem.00068-26-s0001.pdf]

Supplementary Materials

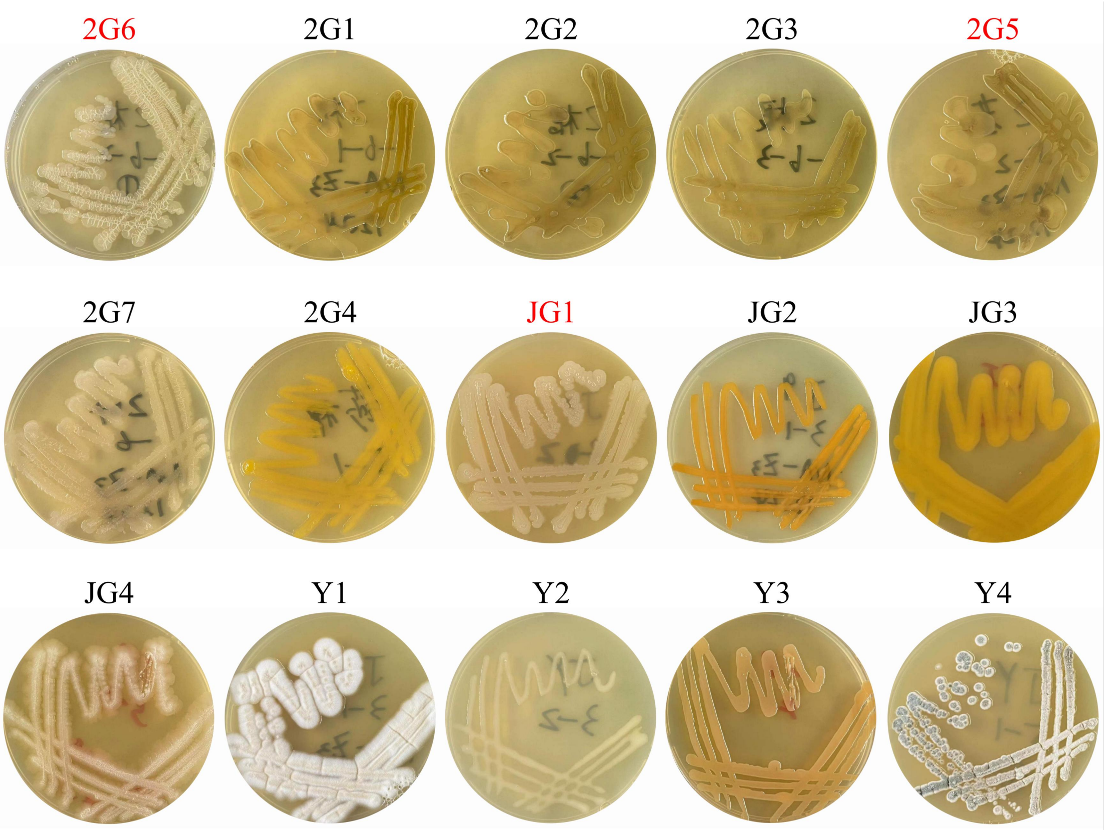

Fig. S1. 15 purified strains were obtained by preliminary separation.

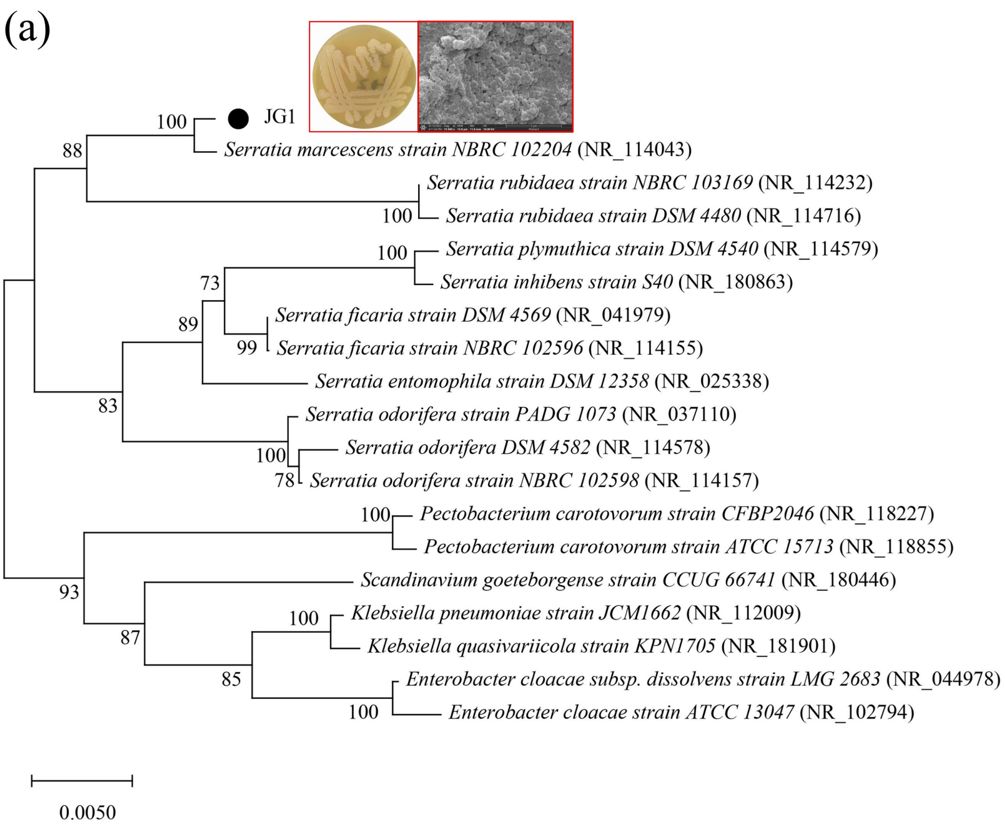

(b)

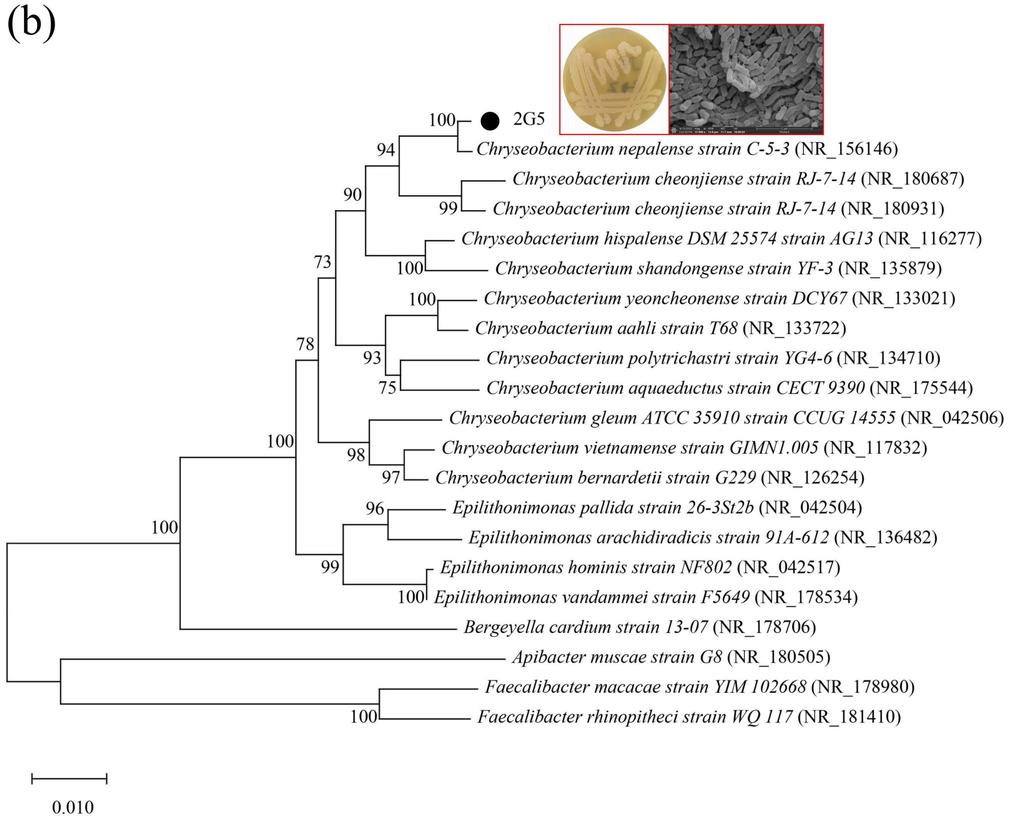

(c)

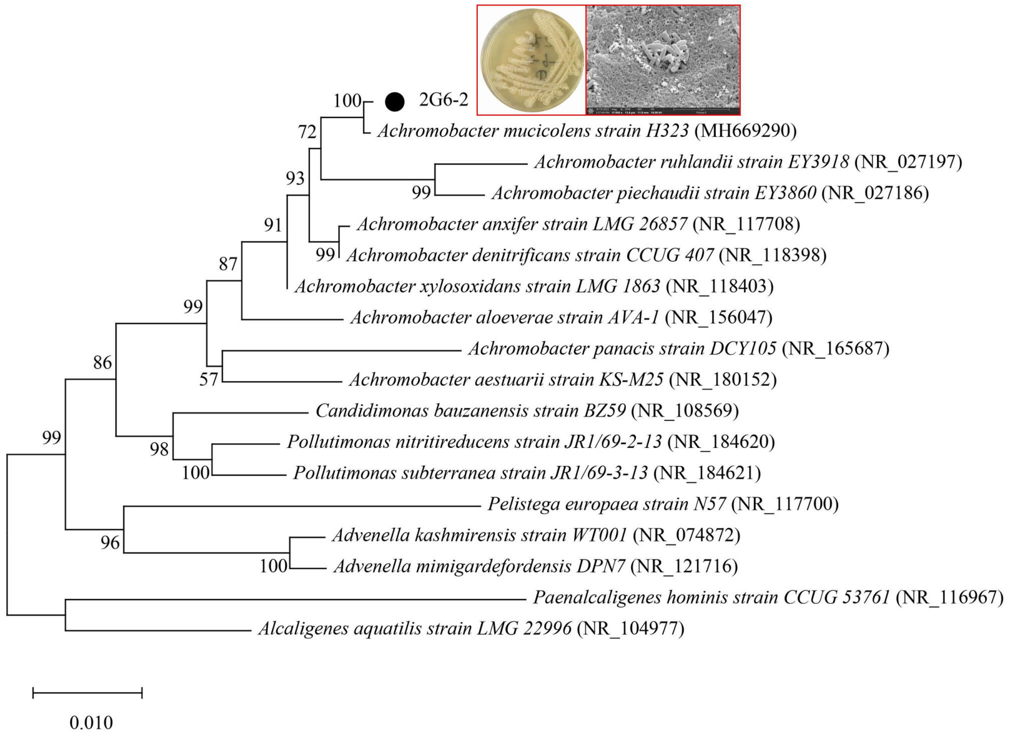

Fig. S2. Phylogenetic tree analysis of strains JG1 (a), 2G5 (b) and 2G6 (c) based on the partial 16S rDNA gene sequences.

Table S1. Colony diameters of 15 purified strains on plates containing Pb^{2+} (400 mg/L) and Cd^{2+} (50 mg/L).

Strains	Growth on heavy metal plate	Colony diameter (cm)
2G6	+	1.20
2G1	—	—
2G2	+	1.15
2G3	—	—
2G5	+	1.32
2G7	—	—
2G4	—	—
JG1	+	1.30
JG2	—	—
JG3	—	—
JG4	—	—
Y1	+	1.02
Y2	—	—
Y3	—	—
Y4	—	—
Y5	—	—

"+" represents that strain grows normally on the plate, and "-"indicates that strain no growth normally or the growth diameter is <1.0 cm on the plate.

Table S2. Physiological and biochemical characteristics of 5 strains after re-screening

Strains	IAA production (μg/mL)	Phosphate solubilization (D/d)		Siderophore production (D/d)		Nitrogen fixation	Citric acid production
JG1	15.12 ± 0.21	+	2.20	+	3.40	+	+
2G5	17.35 ± 0.25	+	2.50	+	4.00	+	+
2G6	13.50 ± 0.19	+	1.45	+	2.20	+	+
2G2	5.12 ± 0.22	+	1.00	-	1.10	-	-
Y1	6.35 ± 0.26	-	0.95	-	1.15	-	-

Table S3. The background values of initial soil and contaminated soil in the pot experiment.

Index	Initial soil	Contaminated soil
TN	1.25 ± 0.03	-
AN	212.32 ± 1.20	184.16 ± 5.12
TP	1.11±0.02	-
AP	124.05 ±0.32	107.81 ±5.11
T_Cd	2.69 ± 0.44	72.67± 2.04
Y_Cd	0.67 ± 0.13	50.67 ± 0.13
T_Pb	75.16 ± 5.18	315.16 ± 5.18
Y_Pb	36.71 ± 1.90	140.27 ± 3.10

TN, TP, AN, and AP represent total nitrogen, total phosphorus, hydrolyzed nitrogen and available phosphorus, respectively. T-Cd and T-Pb represent the total amount of Cd and Pb in soil, respectively. Y-Cd and Y-Pb represent the available contents of Cd and Pb in soil, respectively. Values in the table represent means ± standard deviation

(SD).

Soil DNA extraction, PCR amplification, and high-throughput gene sequencing

The complete genomic DNA was extracted from 0.25 g of rhizosphere soil collected from each pot using the e.z n.a.® Soil DNA Kit (Omega Bio-Tek, Norcross, GA, USA) referring to the instructions of manufacturer (1). Nanodrop RND-2000 (NanoDrop Technologies, Wilmington, DE, United States) was used for quantitative analysis of extracted DNA, while qualitative analysis was performed using agarose gel (1%) electrophoresis (2). The high-quality DNA fragments weres amplified by PCR using primers 338F (5'-ACTCCTACGGGAGCAGCAG-3') and 806R (5'-GGACTACHGGGTWTCTAAT-3') (3), and MiSeq sequencing was performed on the V3-V4 area of bacterial 16S rRNA gene. The PCR reaction mixture (25 µL) contained 2 µL of DNA extract (5-20 ng), 1 µL of each primer (10 µm), 12.5 µL of 2× Premix Taq™ (TaKaRa. Bio Inc. Shiga, Japan), and 9 µL of dd H_2O. The thermal cycling stages during PCR were as follows: initial denaturation (3 min, 95°C), followed by 35 cycles of denaturation (30 s, 95°C) and annealing (30 s, 55°C), and a final step of DNA extension (10 min, 72°C). After amplification, PCR products obtained from same sample were mixed. The quality of mixed amplicon was assessed by agarose gel (2%) electrophoresis and the high-quality product was recovered from the gel using an AxyPrepDNA gel recovery kit (Axygen Biosciences, USA). Based on the initial quantitative results obtained from electrophoresis, the high-quality PCR products were further assessed using QuantiFluorTM-ST blue fluorescence quantitative system (Promega company, USA). According to the requirements of sequencing experiment, the DNA samples were quantified, mixed, and then sequenced using Mothur (V.1.36.1). The optimized sequences were obtained from raw sequence data by filtering and removing the chimeric sequences. The process flow was as follows. First, FASTP (v. 0.19.6) was used to remove the low-quality reads from the original sequences, and then FLASH (v. 1.2.11) was used for splicing to obtain longer sequences (4). UPARSE v. 11 was used for operational taxonomic unit (OTU) clustering after quality control splicing, and the chimeras were removed according to 97% similarity to acquire the optimized DNA sequences, which were divided into operational classification units (OTUs) (5). MiSeq sequences of purified amplicons were high-throughput sequenced by Guangzhou Jidi' ao Technology Service Co., Ltd (Guangzhou, China) using Illumina® MiSeq sequencer (Illumina, San Diego, CA, United States).

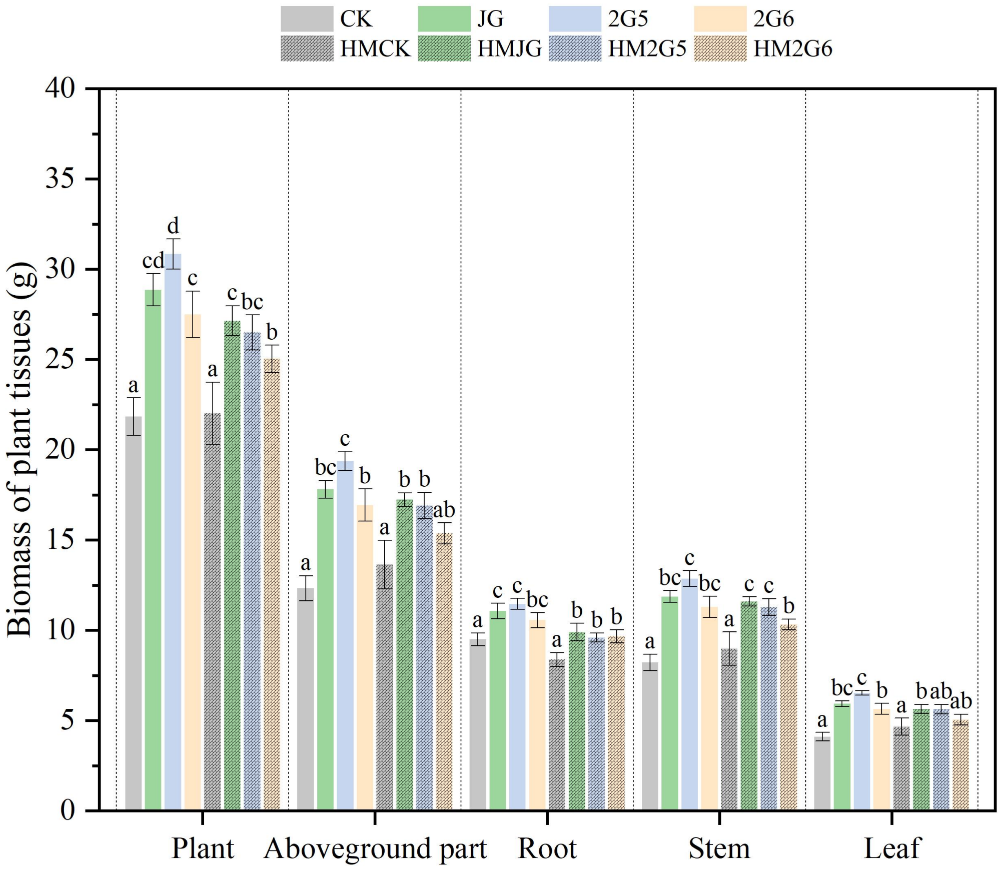

Fig. S3. Effects of different treatments on the growth of A. *fruticosa* L. Plant, Aboveground, Root, Stem, and Leaf in the abscissa represent the biomass of plant, aboveground, root, stem and leaf, respectively. Different lowercase letters obver bars indicate significant differences among different treatments ($p < 0.05$, n = 3, Tukey's test).

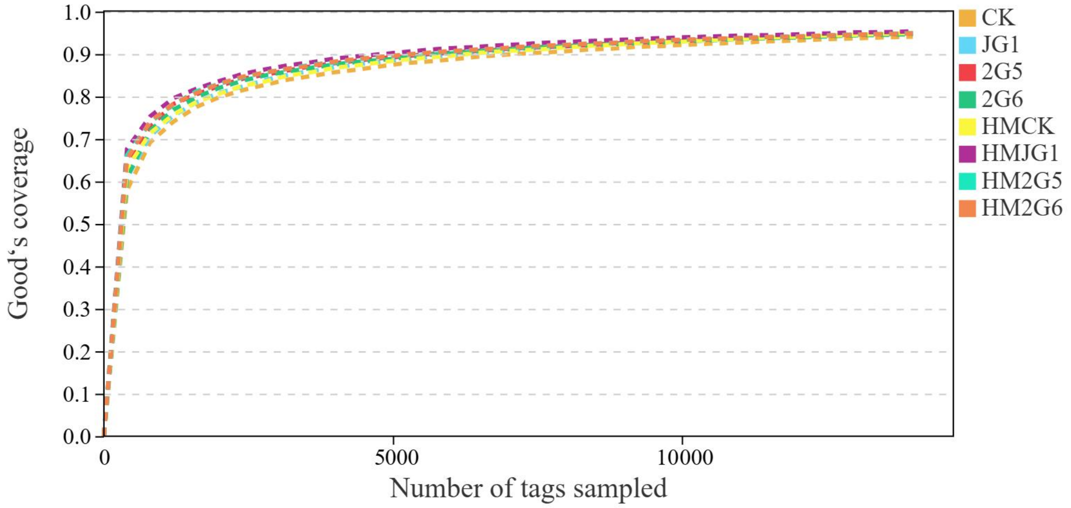

Fig. S4. Variation in the dilution curves of soil bacteria under different treatments.

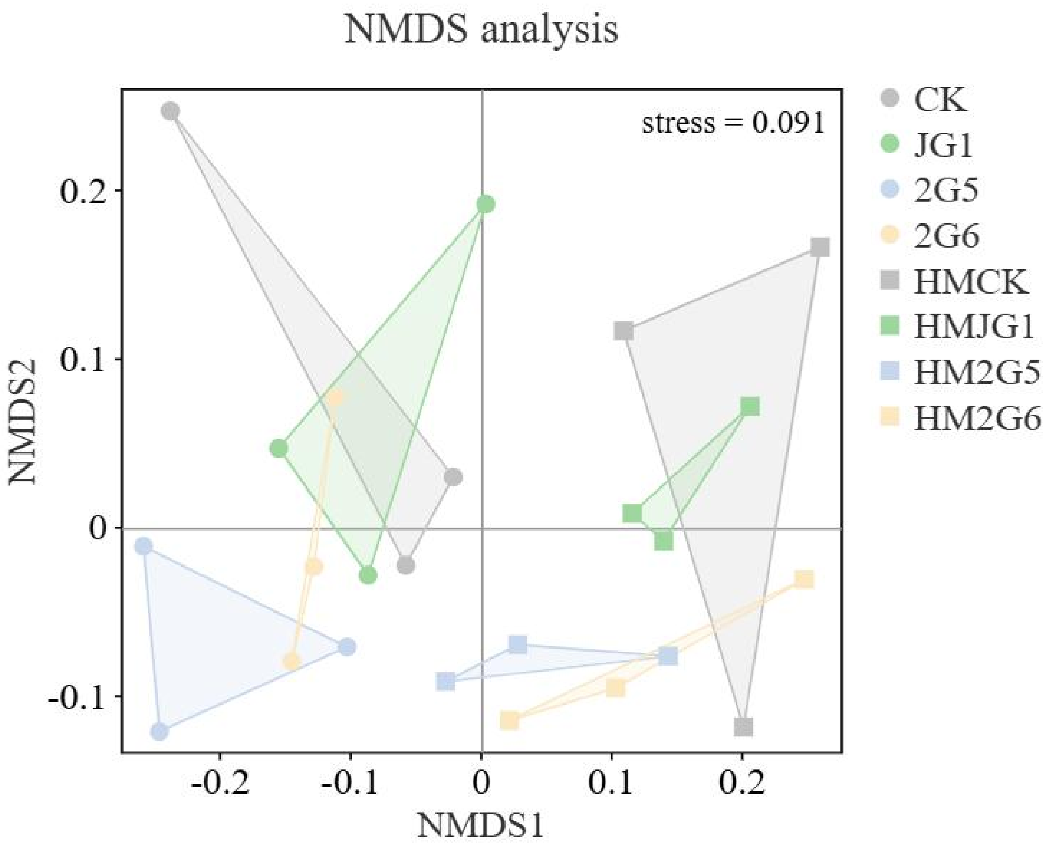

Fig. S5. Nonmetric multidimensional scaling (NMDS) analysis of different treatments of rhizosphere soils based on the Jaccard distance matrix.

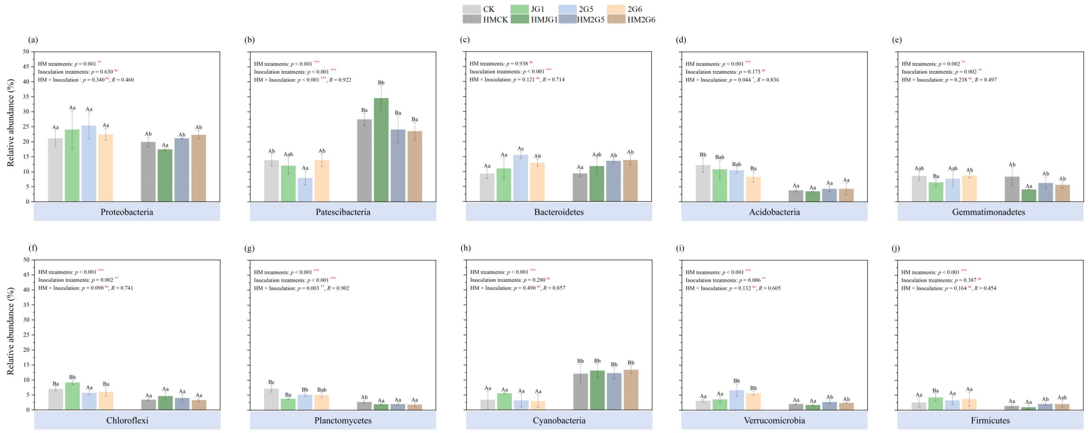

Fig. S6. Effects of different treatments on the top ten species of soil bacterial communities at phylum level. *, ** and *** denote a significant differences between different treatments at $p < 0.05$, $p < 0.01$and $p < 0.001$, respectively, and ns denote no significant difference. Different lowercase letters indicate significant differences between different microbial inoculants under the same soil conditions. Different capital letters indicate significant differences between different soils under the same inoculation conditions.

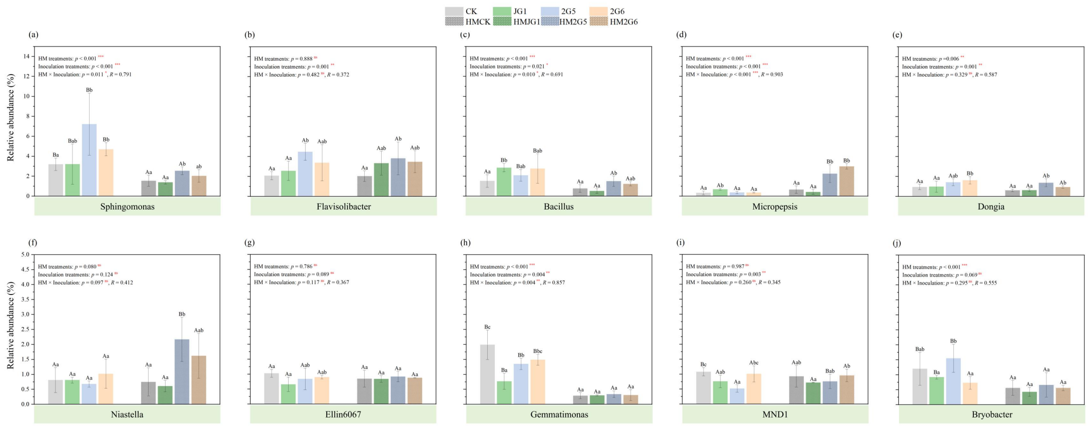

Fig. S7. Effects of different treatments on the top ten species of soil bacterial communities at genus level.*, ** and *** denote a significant differences between different treatments at $p < 0.05$, $p < 0.01$and $p < 0.001$, respectively, and ns denote no significant difference. Different lowercase letters indicate significant differences between different microbial inoculants under the same soil conditions. Different capital letters indicate significant differences between different soils under the same inoculation conditions.

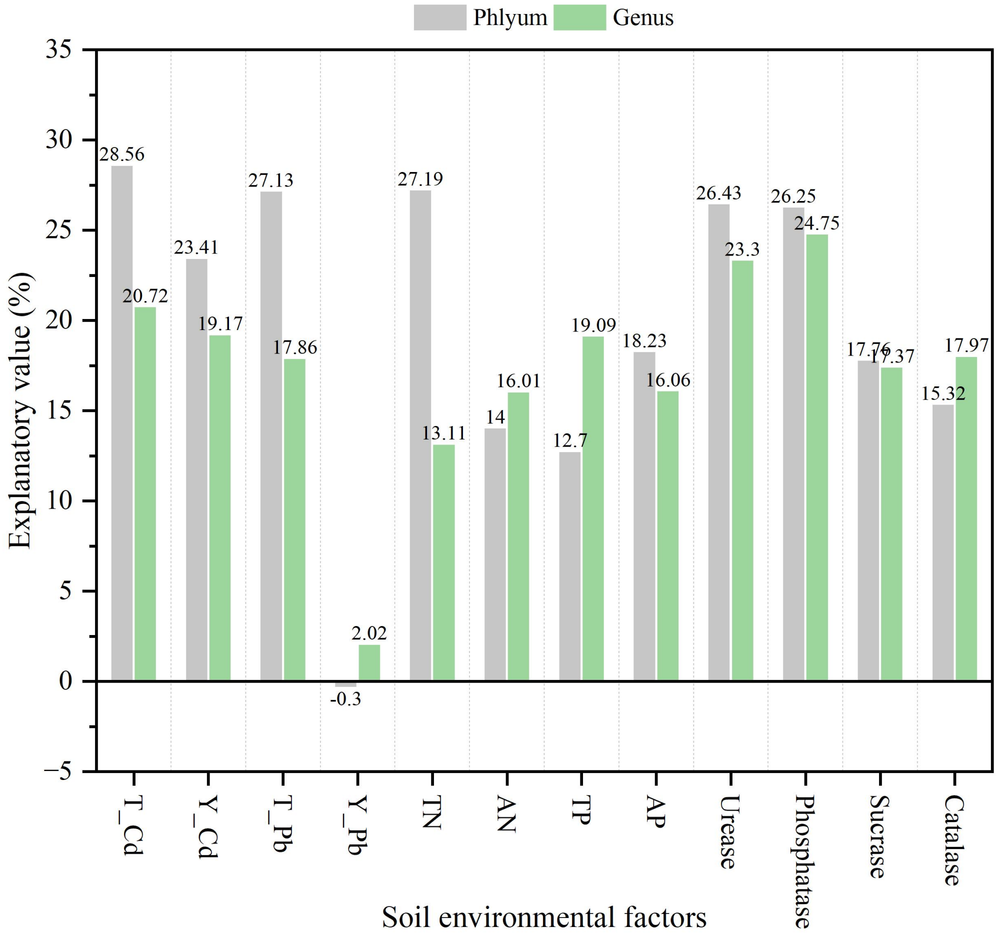

Fig. S8. The contribution degree of environmental factors to the levels of phylum and genus based on Mantel test analysis.

Table S4. Topological parameters of soil bacterial co-occurrence networks.

Network Parameters	CK	TRE	HMCK	HMTRE
**Node**	198	182	136	139
Edge	1343	1002	713	693
Density	0.069	0.061	0.078	0.072
Average_degree	13.566	11.011	10.485	9.971
Average_weighted_degree	7.561	3.911	4.16	2.546
Clustering_coefficient	0.799	0.798	0.821	0.798
**Modularize**	1.129	2.407	1.814	5.905
Positive_edge	69.85%	62.77%	77.89%	67.76%
Negative_edge	30.15%	37.23%	22.11%	32.24%

TRE represents microbial inoculations treatments in initial soil (including JG1, 2G5 and 2G6 treatments), HMTRE represents microbial inoculations treatments in contaminated soil (including HMJG1, HM2G5 and HM2G6 treatments)

Table S5. Top ten key species taxonomic in initial soil under tolerance-promoting bacteria Inoculation.

Nodes_id	Within_module_ connectivities (*Zi*)	Among_module_ connectivities (*Pi*)	Degree	Modularity	Type	Phylum
OTU003391	4.063	0.296	129	4	Module hubs	Proteobacteria
OTU002125	3.896	0.019	104	4	Module hubs	Planctomycetes
OTU003122	3.601	0.638	85	8	Network hubs	Proteobacteria
OTU000326	3.521	0.2	102	6	Module hubs	Patescibacteria
OTU001460	3.338	0.309	69	1	Module hubs	-
OTU000563	3.246	0.313	68	1	Module hubs	Proteobacteria
OTU001060	3.246	0.313	68	1	Module hubs	Cyanobacteria
OTU001225	3.246	0.293	67	1	Module hubs	Verrucomicrobia
OTU000778	3.061	0.213	61	1	Module hubs	Proteobacteria
OTU002086	3.06	0.182	92	6	Module hubs	Chloroflexi
OTU002243	3.06	0.182	92	6	Module hubs	Patescibacteria
OTU002188	2.969	0.283	63	1	Module hubs	Planctomycetes
OTU001593	2.898	0.087	89	4	Module hubs	Acidobacteria
OTU002619	2.875	0.409	92	2	Module hubs	Planctomycetes
OTU001641	2.842	0.415	118	4	Module hubs	Firmicutes
OTU001704	2.842	0.398	115	4	Module hubs	Acidobacteria
OTU001888	2.842	0.415	118	4	Module hubs	-
OTU001912	2.842	0.415	118	4	Module hubs	Actinobacteria
OTU001940	2.787	0.296	101	4	Module hubs	Planctomycetes
OTU001989	2.787	0.317	103	4	Module hubs	Planctomycetes
OTU002922	2.787	0.317	103	4	Module hubs	Proteobacteria
OTU003088	2.787	0.317	103	4	Module hubs	Planctomycetes
OTU000218	2.772	0.204	88	6	Module hubs	Acidobacteria
OTU001908	2.689	0.583	28	7	Module hubs	Planctomycetes
OTU002210	2.689	0.522	26	7	Module hubs	Acidobacteria
OTU000825	2.676	0.422	115	4	Module hubs	Planctomycetes
OTU001459	2.676	0.422	115	4	Module hubs	Chloroflexi
OTU002293	2.669	0.524	103	2	Module hubs	Chloroflexi
OTU001809	2.657	0.642	76	8	Network hubs	Bacteroidetes
OTU001064	2.657	0.156	83	6	Module hubs	Proteobacteria
OTU001229	2.657	0.156	83	6	Module hubs	Patescibacteria
OTU001295	2.657	0.156	83	6	Module hubs	Proteobacteria
OTU000041	2.541	0.026	75	6	Module hubs	Patescibacteria
OTU000578	2.541	0.052	76	6	Module hubs	Actinobacteria
OTU001086	2.541	0.16	81	6	Module hubs	Proteobacteria
OTU001915	2.541	0.16	81	6	Module hubs	Proteobacteria
OTU001091	2.51	0.025	79	4	Module hubs	Proteobacteria

"-"represents other species.

Table S6. Top ten key species taxonomic in contaminated soil under tolerance-promoting bacteria Inoculation.

Nodes_id	Within_module_connectivities (*Zi*)	Among_module_connectivities (*Pi*)	Degree	Modularity	Type	Phylum
OTU001565	3.967	0.062	63	10	Module hubs	Proteobacteria
OTU001271	3.574	0.417	51	9	Module hubs	Verrucomicrobia
OTU002171	3.551	0.124	61	10	Module hubs	Proteobacteria
OTU002197	3.533	0.123	138	11	Module hubs	Proteobacteria
OTU000684	3.447	0.035	57	10	Module hubs	Planctomycetes
OTU002278	3.344	0.128	59	10	Module hubs	-
OTU003516	3.344	0.1	58	10	Module hubs	Patescibacteria
OTU001960	3.332	0.303	152	11	Module hubs	Planctomycetes
OTU002027	3.24	0.365	70	10	Module hubs	Firmicutes
OTU003150	3.24	0.365	70	10	Module hubs	Bacteroidetes
OTU002641	3.185	0.442	79	8	Module hubs	-
OTU005103	3.136	0.307	65	10	Module hubs	Acidobacteria
OTU001933	3.131	0.132	128	11	Module hubs	Verrucomicrobia
OTU000252	3.032	0.072	54	10	Module hubs	Acidobacteria
OTU001416	3.032	0.256	61	10	Module hubs	Chloroflexi
OTU002302	3.032	0.105	55	10	Module hubs	Chloroflexi
OTU001035	3.01	0.017	117	11	Module hubs	-
OTU001430	3.009	0.585	96	8	Module hubs	Patescibacteria
OTU001695	3.009	0.585	96	8	Module hubs	Proteobacteria
OTU001058	2.928	0.107	54	10	Module hubs	Patescibacteria
OTU001870	2.928	0.107	54	10	Module hubs	Proteobacteria
OTU001285	2.889	0.299	138	11	Module hubs	Verrucomicrobia
OTU002156	2.889	0.067	117	11	Module hubs	Proteobacteria
OTU000350	2.849	0.018	113	11	Module hubs	Bacteroidetes
OTU000797	2.832	0.568	92	8	Module hubs	-
OTU001326	2.832	0.568	92	8	Module hubs	Patescibacteria
OTU001701	2.824	0.109	53	10	Module hubs	-
OTU002101	2.824	0.195	56	10	Module hubs	Proteobacteria
OTU000005	2.796	0.622	52	1	Network hubs	Patescibacteria
OTU001945	2.769	0.018	111	11	Module hubs	Proteobacteria
OTU001155	2.744	0.599	95	8	Module hubs	Planctomycetes
OTU001640	2.744	0.599	95	8	Module hubs	Patescibacteria
OTU002246	2.744	0.599	95	8	Module hubs	Proteobacteria
OTU006648	2.742	0.613	20	7	Module hubs	Patescibacteria
OTU000873	2.72	0.111	52	10	Module hubs	Gemmatimonadetes
OTU000314	2.688	0.018	109	11	Module hubs	Bacteroidetes
OTU000564	2.648	0.018	108	11	Module hubs	Patescibacteria
OTU001312	2.648	0.285	129	11	Module hubs	Acidobacteria
OTU000704	2.616	0.145	52	10	Module hubs	Bacteroidetes

OTU001387	2.616	0.226	55	10	Module hubs	Patescibacteria
OTU002108	2.616	0.078	50	10	Module hubs	Patescibacteria
OTU002527	2.616	0.04	49	10	Module hubs	Patescibacteria
OTU000580	2.608	0.019	107	11	Module hubs	Proteobacteria
OTU001199	2.568	0.501	75	8	Module hubs	Proteobacteria
OTU001528	2.568	0.501	75	8	Module hubs	-
OTU003429	2.568	0.501	75	8	Module hubs	-
OTU001176	2.527	0.019	105	11	Module hubs	Chloroflexi
OTU001746	2.527	0.072	108	11	Module hubs	Actinobacteria
OTU001499	2.512	0.041	48	10	Module hubs	Patescibacteria

“-”represents other species.

References

1. Li J, Xu Y, Song QW, Yang J, Xie L, Yu SH, Zheng L. 2021. Polycyclic aromatic hydrocarbon and n-alkane pollution characteristics and structural and functional perturbations to the microbial community: a case-study of historically petroleum-contaminated soil. Environ Sci Pollut Res Int 28 (9), 10589-10602. https://doi.org/10.1007/s11356-020-11301-1
2. Lou JL, Liu M, Gu JL, Liu QH, Zhao L, Ma YS, Wei DZ. 2019. Metagenomic sequencing reveals microbial gene catalogue of phosphinothricin-utilized soils in South China. Gene 711, 143942. https://doi.org/10.1016/j.gene.2019.143942
3. Ma H, Wei MY, Wang ZR, Hou SY, Li XD, Xu H, 2020. Bioremediation of cadmium polluted soil using a novel cadmium immobilizing plant growth promotion strain *Bacillus* sp. TZ5 loaded on biochar. J Hazard Mater 388, 122065. https://doi.org/10.1016/j.jhazmat.2020.122065
4. Callahan BJ, McMurdie PJ, Rosen MJ, Han AW, Johnson AJ, Holmes SP. 2016. DADA2: High-resolution sample inference from Illumina amplicon data. Nat Methods 13 (7), 581-583. https://doi.org/10.1038/nmeth.3869
5. Edgar RC, Haas BJ, Clemente JC, Quince C, Knight R, 2011. UCHIME improves sensitivity and speed of chimera detection. Bioinformatics 27 (16), 2194-2200. https://doi.org/10.1093/bioinformatics/btr381
